# Recent Progress of Electrode Materials for Flexible Perovskite Solar Cells

**DOI:** 10.1007/s40820-022-00859-9

**Published:** 2022-04-30

**Authors:** Yumeng Xu, Zhenhua Lin, Wei Wei, Yue Hao, Shengzhong Liu, Jianyong Ouyang, Jingjing Chang

**Affiliations:** 1 State Key Discipline Laboratory of Wide Band Gap Semiconductor Technology, School of Microelectronics, Xidian University, 2 South Taibai Road, Xi’an, 710071 People’s Republic of China; 2 Advanced Interdisciplinary Research Center for Flexible Electronics, Xidian University, 2 South Taibai Road, Xi’an, 710071 People’s Republic of China; 3 Key Laboratory of Applied Surface and Colloid Chemistry, Ministry of Education, Institute for Advanced Energy Materials, School of Materials Science and Engineering, Shaanxi Normal University, Xi’an, 710119 People’s Republic of China; 4 Department of Materials Science and Engineering, National University of Singapore, 7 Engineering Drive 1, Singapore, 117574 Singapore

**Keywords:** Flexible electrode, Flexible perovskite solar cell, Carbon nanomaterials, Metallic nanostructures, Conductive oxide

## Abstract

Flexible perovskite solar cells (FPSCs) have attracted enormous interest in wearable and portable electronics due to their high power-per-weight and low cost. Flexible and efficient perovskite solar cells require the development of flexible electrodes compatible with the optoelectronic properties of perovskite. In this review, the recent progress of flexible electrodes used in FPSCs is comprehensively reviewed. The major features of flexible transparent electrodes, including transparent conductive oxides, conductive polymer, carbon nanomaterials and nanostructured metallic materials are systematically compared. And the corresponding modification strategies and device performance are summarized. Moreover, flexible opaque electrodes including metal films, opaque carbon materials and metal foils are critically assessed. Finally, the development directions and difficulties of flexible electrodes are given.

## Highlights

- Convincing candidates of flexible transparent electrodes are discussed in detail from the views of fabrication, properties and device performance.
- The progresses of flexible opaque electrodes used in flexible perovskite solar cells are provided.
- The future directions and challenges in developing flexible electrodes are highlighted.

## Graphical abstract

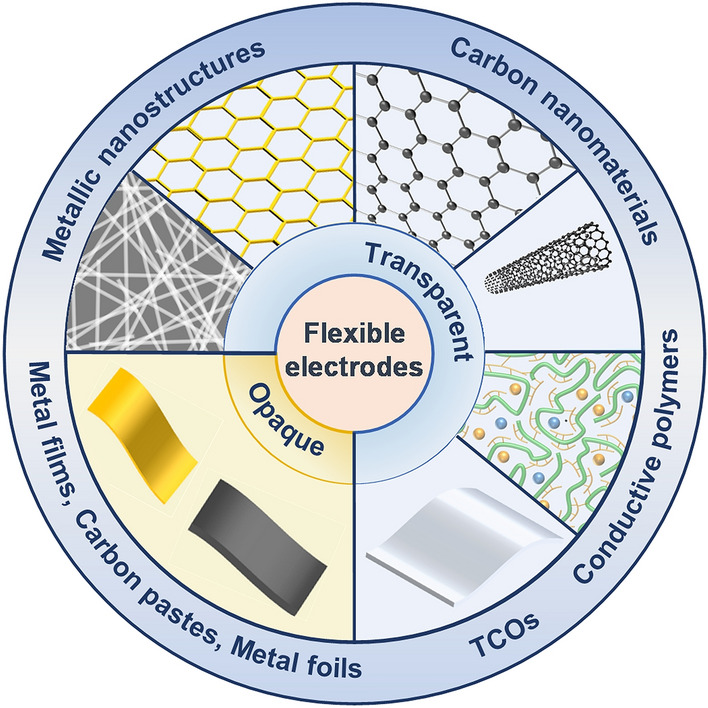

## Introduction

Perovskite materials with excellent optoelectronic properties and simple manufacture are attractive in various optoelectronic applications [1–4]. With the power conversion efficiency (PCE) rushed to 25.7 from 3.8%, perovskite solar cells (PSCs) show great potential in photovoltaic applications [5, 6]. Meanwhile, owing to the growing interest in wearable and self-powered flexible electronics, the demand for flexible perovskite solar cells (FPSCs) is spreading as the perovskite film is advantageous in realizing flexible devices due to the low-temperature processability and intrinsic flexibility. Besides, FPSCs have advantages of low cost due to the roll-to-roll processability, which can shorten the fabrication time, save materials and enable large-scale device manufacturing. And the light weight and easy package of FPSCs also account for the lessened cost in transportation and installation. Moreover, FPSCs demonstrate excellent power-per-weight among the various flexible solar cells like copper indium gallium selenide (CIGS), cadmium telluride (CdTe) and organic solar cells, which is favorable in the application of wearable smart electronics [7–9]. Additionally, FPSCs can also achieve stretchability, self-healing ability, biocompatibility and biodegradability by selecting appropriate materials, which can broaden their application prospects and be environmentally friendly [10–12].

Generally, standard planar FPSCs comprise the flexible substrate, bottom electrode, charge transporting layers, perovskite film and top electrode. To produce high-performance FPSCs, the flexible substrate should have high flexibility to sustain repeated bending processes, broad optical transparency to achieve high light utilization, good thermal stability to endure the annealing process during subsequent films deposition, and good hermeticity to avoid moisture invasion. Flexible substrates commonly used in FPSCs are polymers like polyethylene terephthalate (PET) and polyethylene naphthalate (PEN) [13, 14]. Since the polymer substrates are usually sensitive to temperature, low-temperature processability of charge transporting layer is required for the application in FPSCs [15–18]. For perovskite film, proper doping with polymers or self-crosslinked materials could facilitate perovskite grain growth and prevent the formation of cracks, which is beneficial to achieve high-quality and robust perovskite films in FPSCs [19–21]. Moreover, optimizing the structure via lessening the substrate thickness or placing a capping layer above the device could shift the perovskite film to the mechanical neutral plane, enabling robust FPSCs [19, 22].

In a typical bottom-illuminated FPSCs, the bottom electrode (also window electrode) is transparent and plays a significant role in achieving high-performance FPSCs, because the incident light needs to pass through the transparent electrode and ultimately be absorbed by the perovskite layer, and the photo-generated charges must be collected by the electrode. Transparent electrodes commonly used in FPSCs include transparent conductive oxides (TCOs), conductive polymers, carbon nanomaterials, and metallic nanostructures. The top electrode (also back electrode) could be opaque or transparent. The noble metal films like Au and Ag are the mostly used opaque top electrode. In addition, opaque carbon materials are low-cost substitutes to the expensive metal electrode, and could enable hole transport layer (HTL)-free devices. When transparent top electrode and transparent bottom electrode are applied simultaneously, semitransparent FPSCs could be achieved [23, 24]. Researchers proved that the chemical composition of perovskite materials could be changed by different underlying materials, proving the importance of bottom electrode [25]. Moreover, the mechanical properties of flexible electrodes are significant to flexible devices. The device performance deterioration of FPSCs during bending process is usually caused by flexible electrodes due to the decrease of conductivity and film detachment [26]. For example, the cracks formed in brittle indium tin oxide (ITO) electrode during bending process often leads to device deterioration [26]. Thus, the development of high-performance flexible electrodes is the key in manufacturing highly efficient FPSCs.

In this review, promising candidates for flexible electrodes in FPSCs are categorized and assessed critically. According to their optical properties, we divide the flexible electrodes into flexible transparent electrodes and flexible opaque electrodes. In Sect. 2, we review flexible transparent electrodes including TCOs, conductive polymer, carbon nanomaterials (*e.g.,* carbon nanotubes (CNTs), graphene), and metallic nanostructures (*e.g.,* metal nanowires, metal meshes, ultrathin metal films (UTMFs, about 10 nm thick), oxide/metal/oxide (OMO) and metal foil). We comprehensively compare these flexible transparent electrodes from the aspects of optoelectronic performance (optical transparency and electrical conductivity), mechanical properties (flexibility and robustness), and ease of fabrication. Moreover, we summarize their limitations in the application of FPSCs when employed as bottom electrode or top electrode. The corresponding modification methods, such as additive engineering, interface modification and advanced fabrication methods are also discussed. In Sect. 3, we review flexible opaque electrodes including metal films (60–100 nm thick), opaque carbon materials, and metal foils (over 100 µm thick). In the end, the difficulties and future trends of flexible electrodes in realizing highly efficient and flexible FPSCs are provided.

## Flexible Transparent Electrodes

To be a good candidate in the application of FPSCs, flexible transparent electrode must possess high optical transparency and electrical conductivity for sufficient light utilization and adequate charge collection, which are directly related to the efficiency of FPSCs. They must also be robust and durable to maintain their optoelectrical properties during deformation and bending for mechanically stable FPSCs. Different flexible transparent electrodes employed in FPSCs are summarized in Table 1 with their optoelectronic parameters and device performances. Moreover, high chemical stability and low-cost preparation process of flexible transparent electrodes are all required to produce long-term stable and cost-effective FPSCs. Previous studies identified that TCOs, conductive polymer, carbon nanomaterials and metallic nanostructures are promising candidates in achieving efficient and stable FPSCs. In this section, their fabrication methods, physical properties, modification strategies are thoroughly reviewed, and their applications as bottom or top electrode in FPSCs are also discussed.

**Table 1:** Summary of flexible transparent electrodes

Substrate/electrode	Sheet resistance (Ω/sq)	Optical transmittance (%)	Flexibility of the related device	Efficiency (%)	Refs.
Glass/ITO	10 [18]	86 [18]	–	24.4 [161]	–
PET/ITO	10–15 [162]	73.1 [162]	20,000 tension-only cycles at r = 5 mm (81% of initial PCE left)	21.0 [144]	–
PEN/ITO	14	78	2,000 bending cycles at r = 10 mm (93.2% of initial PCE left)	21.10	[35]
Willow glass/ITO	12 [162]	85 [162]	Bending radius at centimeter scale [162]	19.72 [163]	–
PET/PEDOT:PSS	24	84.6	5,000 bending cycles at r = 3 mm (85% of initial PCE left)	19.0	[74]
PET/PEDOT:PSS	234.3	> 80	10,000 bending cycles at r = 0.5 mm (no clear efficiency decrease)	17.03	[22]
PDMS/PH1000	40	–	5,000 bending cycles r = 2 mm (no clear efficiency decrease)	15.01	[19]
NOA 63/PEDOT:PSS	110	91 (NOA 63) 93.3 (PEDOT:PSS)	1000 bending cycles r = 1 mm (90% of initial PCE left)	10.83	[65]
PI-SWNT/MoO_x_	82	80 (at 700 nm)	10,000 folding cycles r = 0.5 mm (no efficiency decrease)	15.2	[91]
PET/2-layed graphene	290 ± 17	87.3 (at 500 nm)	2,000 bending cycles r = 4 mm (86% of initial PCE left)	11.9	[95]
PEN/graphene/MoO_3_	552.0	97	5, 000 bending cycles r = 2 mm (85% of initial PCE left)	16.8	[96]
PDMS/APTES/TFSA-doped graphene	116 ± 49	96.8	5,000 bending cycles r = 8 mm (75% of initial PCE left)	18.3	[77]
PDMS/graphene (top)	396 ± 23	92.5	1,000 bending cycles r = 4 mm (less than 80% of initial PCE left)	15	[98]
Polymer/AgNWs /graphene	8.06	88.3 (at 550 nm)	–	10.419	[110]
PEN/orthogonal AgNWs	15.7	92.3	1,000 bending cycles r = 5 mm (80% of initial PCE left)	12.85	[8]
PET/Ag-mesh/PH1000	3	82–86	5,000 bending cycles r = 5 mm (95.4% of initial PCE left)	14.0 [126] 18.1 [164]	[126]
PET/PDMS/Ag grids	7.9	> 85	1,000 bending cycles r = 6 mm (60% of initial PCE left)	18.49	[130]
PET/Au-mesh /PEDOT:PSS	18.0	> 80	10,000 bending cycles r = 0.5 mm (no clear efficiency decrease)	13.6 (1.2 cm^2^)	[22]
Cellophane paper/ TiO_2_/ultrathin Ag/TiO_2_	9.5	82.1	1,000 bending cycles r = 1 mm (95.8% of initial PCE left)	13	[137]

### Transparent Conductive Oxides

The conventional electrode material TCOs are widely applied in PSCs, including ITO, fluorine doped tin oxide (FTO), indium zinc oxide (IZO) [25, 27], aluminum doped zinc oxide (AZO) [25, 28] and W-doped In_2_O_3_ (IWO) [29]. Among the above-mentioned TCOs, the manufacturing temperature of FTO is too high to apply on polymer substrates. Specifically, FTO is deposited at substrate temperature above 350 ℃ [30, 31], while IZO could be deposited at 100 ℃ [32], and ITO, AZO and IWO can be prepared at room temperature [28, 29, 33]. For AZO, according to the researches of Dou et al., they found evidence that AZO has chemical interactions with perovskite films, which results in decreased performance of FPSCs [25, 28]. For comparison, IZO has a lower sheet resistance and the related FPSCs show a higher FF, while ITO-based FPSCs have better short-circuit current density (*J*_sc_) due to the higher optical transmittance [25]. Additionally, IZO films encounter the same problem as ITO films when applied to FPSCs: the breakage of the brittle IZO layer during bending at a small radius [27, 34]. Most recently, Kim et al. prepared IWO via room-temperature arc plasma ion plating method to replace ITO in FPSCs. Attributing to higher optical transmittance especially in near-infrared range and higher work function (4.85 eV) compared to ITO (4.65 eV), the IWO-based FPSCs showed a higher power conversion efficiency of 11.33% than ITO-based FPSCs (10.6%) [29].

**Fig. 1:**
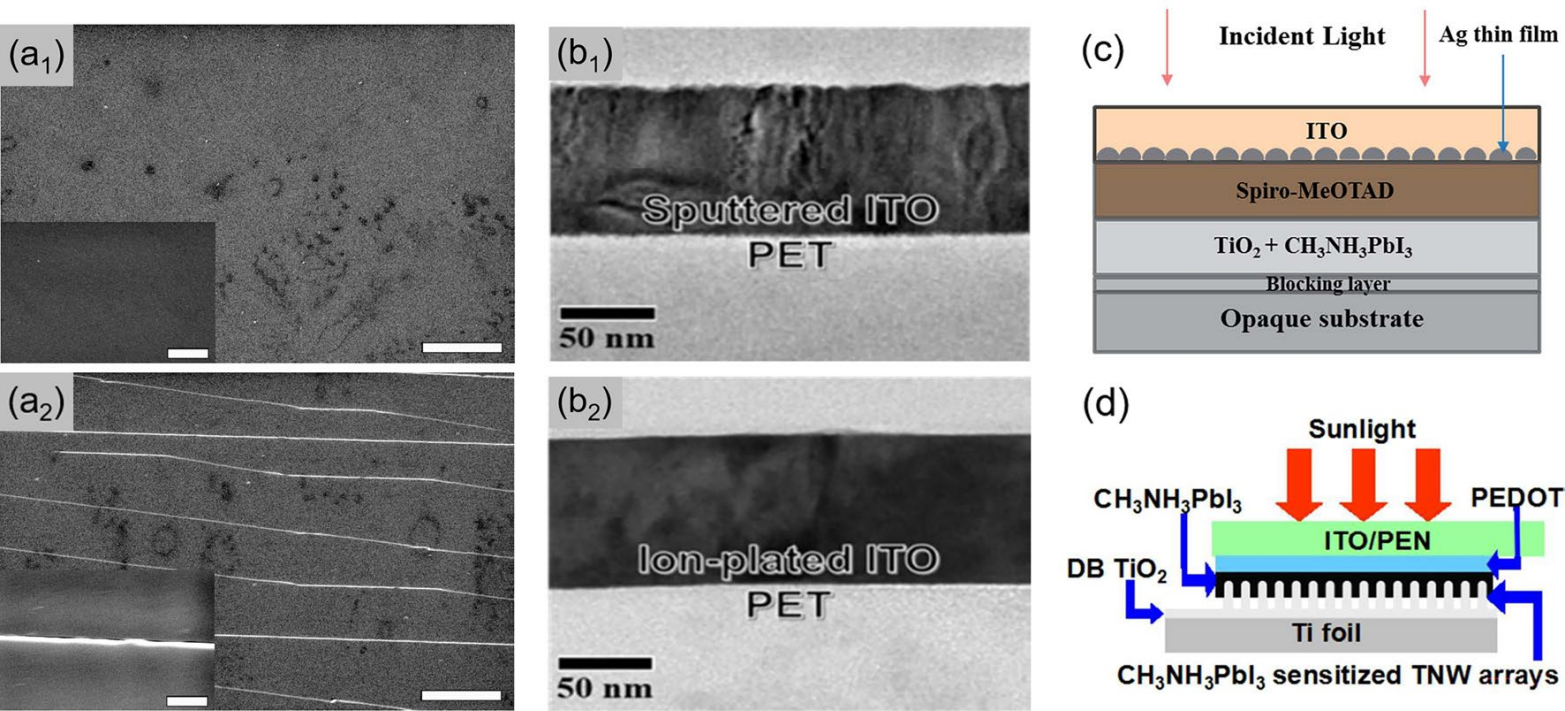
SEM images of PET/ITO substrate before (**a**_**1**_) and after (**a**_**2**_) 2200 bending cycles, and the scale bars are 50 μm (main) and 5 μm (inset), respectively. Reproduced with permission from Ref. [43]. Copyright 2015, Royal Society of Chemistry. Cross-section images about the sputtered ITO film (**b**_**1**_) and ion-plated ITO film (**b**_**2**_). Reproduced with permission from Ref. [52]. Copyright 2018, Royal Society of Chemistry. **c** Device structure of FPSCs with sputtered ITO top electrode. Reproduced with permission from Ref. [58]. Copyright 2015, Royal Society of Chemistry. **d** Device schematic of FPSCs with laminated ITO/PEN top electrode. Reproduced with permission from Ref. [59]. Copyright 2016, Royal Society of Chemistry

Nevertheless, the most widely used transparent electrode in FPSCs is ITO, which could be ascribed to the low-temperature fabrication process, chemical stability against perovskite materials, high optical transparency, well-matched band structure, and most importantly the mature mass production process. And the most efficient FPSCs with recorded PCE over 20% are usually based on commercially available polymer/ITO substrates [35–38]. Despite the extensive usage, the ITO electrode is far from ideal for FPSCs applications, which can be ascribed to five serious issues: (1) The ideal FPSCs are expected to maintain about 90% of the original efficiency after bending 1000 cycles at a radius of 4 mm to meet the needs of flexible and wearable electronics [39]. It is hard for ITO-based FPSCs to approach this requirement due to the inadequate flexibility and the breakage property. The cracks in ITO film and the increased sheet resistance during flexing cycles lead to the decline of device performance [26, 40–44]. The research of Kelly’s group compared different electrodes on PET substrates via bending test and found obvious cracks on metal oxide electrode (Fig. 1a1-a2) with a sharp rise of sheet resistance while negligible change on poly(3,4-ethylenedioxythiophene):poly(styrenesulfonate) (PEDOT:PSS) electrode [43]. (2) The scarcity of indium and the costly vacuum deposition techniques such as sputtering, evaporation and electroplating make ITO the most expensive component in PSCs (about 50–60% of the total material cost), limiting the potential for mass production [45]. (3) The high surface roughness of ITO films ascribed to the resputtering effect will cause current leakage and poor flexibility. (4) The poor transparency in near-infrared region decreases the light utilization. (5) The chemical stability problem when being exposed to PEDOT:PSS, acid and base [46–49].

Continuous improvements of ITO in terms of optical, electrical, and mechanical properties are in progress. Reducing the thickness enables better flexibility for substrates and less fracture during the bending test, while at the expense of higher pristine resistance. With the decreased ITO thickness from about 400 to 160 nm, Liu and co-workers prepared ITO-based FPSCs with good mechanical durability at a bending radius as small as 4 mm [50]. Besides, the photoelectrical quality of ITO electrode can be improved by the deposition method. The ITO films processed by direct current sputtering at room temperature have an amorphous structure as shown in Fig. 1b1, which causes high sheet resistance, low optical transmittance, rough surface and insufficient flexibility [33, 51, 52]. Recently, a high-performance ITO film on PET substrate with smooth surface, low sheet resistance of 15.75 Ω/sq and optical transmittance of 85.88% was reported by Kim and co-workers via an in-line type vertical plasma arc ion plating method. The high crystallinity and adhesion caused by high-energy ITO ions lead to smoother ITO surface by avoiding the resputtering effect as shown in Fig. 1b2. Consequently, the FPSCs displayed a high efficiency of 16.8% with the application of this ion plated ITO films [52]. Moreover, the annealing temperature also matters. With the annealing temperature up to 300 ℃, the ITO films on colorless polyimide (CPI) substrates demonstrated a lower sheet resistance than on PET substrates which are more sensitive to temperature due to the activation of Sn dopant and the crystallization of ITO layer [33]. In addition, structural engineering optimization of ITO electrode is also beneficial to improve light utilization. Zhu et al. developed a moth-eye-inspired structured ITO electrode, which can couple photons into the perovskite layer for enhanced broadband light harvesting, especially at longer wavelengths [53].

Compared to the widespread use as bottom electrode, there is less literature on the application of TCOs as top electrode, which may be due to the difficulty in fabricating high-quality TCO films without damaging the under layer in FPSCs. The sputtering process and high-temperature environment during the fabrication of TCO films are damaging to the perovskite layer and organic charge transporting layer like [2,2,7,7-tetrakis(N,N-di-p-methoxyphenyl-amine) 9,9-spirosbifluorene] (spiro-OMeTAD). A pre-deposited buffer layer is effective to protect the underlying films from the damage of high energy particles during sputtering, such as atomic layer deposition (ALD)-processed VO_x_ film [54], thermally evaporated MoO_x_ film [55], and solution-processed AZO film [56]. Besides, the novel depositing technique like the linear facing target sputtering method, in which the plasma energy is well confined, is an effective method to deposit TCO materials without damaging the layer underneath [57]. Nevertheless, in flexible devices, only a few studies have applied TCO materials as top electrode. Via direct current (DC) sputtering at room temperature, Lee et al. applied 200 nm thick ITO as a transparent top electrode in FPSCs. The amorphous ITO film demonstrated insufficient mechanical properties and high sheet resistance, which may result from the room temperature fabrication process without further annealing. By growing an ultrathin Ag seed layer before ITO film deposition (Fig. 1c), they obtained Ag-embedded ITO transparent electrode with enhanced crystallinity, improved flexibility and reduced sheet resistance from 35.48 to 7 Ω/sq. The inferior efficiency (11.01%) is probably caused by the inadequate light transmittance of Ag-embeded ITO electrode [58]. Moreover, there are studies that avoid the deposition of ITO layer by applying the commercially available ITO/PEN substrate. Xiao et al. assembled PEDOT:PSS/ITO/PEN (PEDOT:PSS has been electrodeposited on the ITO/PEN substrate) onto the Ti foil/titanium dioxide nanowire substrate with the assistance of binder clips. Then the perovskite precursor solution was injected from the gap to complete the FPSCs as shown in Fig. 1d, and the corresponding FPSC exhibited a PCE of 13.07% [59].

Section 2.1 has briefly reviewed several transparent TCO electrodes and summarized the main concerns when applying ITO in FPSCs, including inferior mechanical flexibility, high fabrication cost, high surface roughness, inadequate light transmittance in long wavelength region and chemical stability problems. Nevertheless, ITO with excellent optical and electrical properties is the most popular flexible transparent electrode in FPSCs, especially as the bottom electrode. However, the application of ITO electrode as a top electrode is usually difficult due to the damaging fabrication process or complex procedures which are not suitable for industrial manufacture.

### Conductive Polymer

Despite polymer/ITO substrates being fairly flexible for the application of FPSCs, the fracture of the ITO electrode during a small curvature radius less than 10 mm always causes the failure of devices under bending conditions. Except as an efficient hole transport material [60–64], the conductive polymer PEDOT:PSS is a favorable alternative to ITO as well. PEDOT:PSS has several advantages as an electrode, including solution processability, high optical transmittance and extreme flexibility even stretchability [7, 65]. It is suitable for the roll-to-roll process which is appealing for large-scale manufacture with low production costs. In addition, the tunable work function of PEDOT:PSS enables the application of both cathode and anode. With the modification of polyethylenimine, the PEDOT:PSS electrode possesses a decreased work function from 5.06 to 4.08 eV owing to the formation of the surface dipole, which is more suitable for electron collection [66]. However, the pristine as-cast PEDOT:PSS films yield low electrical conductivity, while the modified PEDOT:PSS layers can improve the conductivity up to 4000 S cm^−1^ with the additive doping like dimethyl sulfoxide (DMSO) and ethylene glycol (EG) or the treatment of acids and ionic liquids [67–69]. Besides, other novel strategies like acid-assisted transfer-printing have also been explored. Fan et al. systematically reviewed the doping strategies of PEDOT:PSS [68]. In this part, various modification methods for PEDOT:PSS are summarized.

**Fig. 2:**
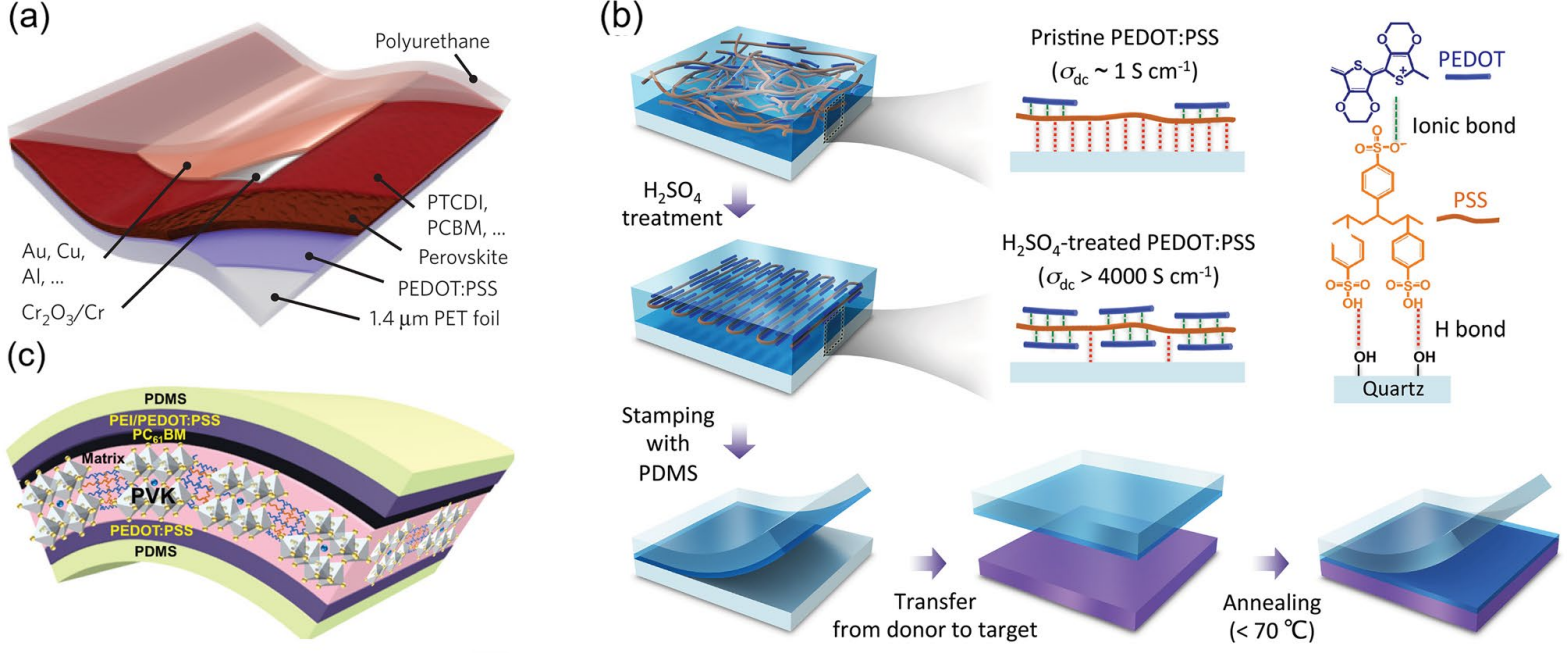
**a** Schematic of FPSCs based on 1.4 μm PET/PEDOT:PSS substrate. Reproduced with permission from Ref. [7]. Copyright 2015, Springer Nature. **b** Illustration of the acid-assisted transfer-printing strategy. Reproduced with permission from Ref. [72]. Copyright 2015, John Wiley and Sons. **c** Schematic of FPSCs with PEDOT:PSS as both cathode and anode. Reproduced with permission from Ref. [19]. Copyright 2019, Royal Society of Chemistry

Dianetti et al. and Poorkazem et al. demonstrated inverted FPSCs by replacing the expensive and fragile ITO electrode with EG modified PEDOT:PSS and DMSO modified PEDOT:PSS on PET substrates, respectively. They both employed the semiconductive PEDOT:PSS as HTL and achieved good mechanical flexibility [42, 43]. In 2015, Kaltenbrunner et al. reported an impressive and promising work by employing DMSO and Zonyl FS-300 doped PEDOT:PSS electrode in FPSCs. They achieved ultrathin (3 μm), highly flexible and air-stable FPSCs on 1.4 μm thick PET substrates with high power-per-weight of 23 W g^−1^ and stabilized efficiency of 12%. In addition, the device realized remarkable endurance even under repeated compression and outstanding long-term stability with the protection of Cr/Cr_2_O_3_/PU caping layer (Fig. 2a) [7]. Park et al. employed DMSO modified PEDOT:PSS as the transparent electrode and electron blocking layer as well on the shape memory substrate Norland Optical Adhesive 63 (NOA63). The fabricated FPSCs have excellent mechanical flexibility and shape recoverability, maintaining 90% of the original PCE after 1000 bending cycles at a small radius of 1 mm and over 50% after 50 cycles of complete crumpling and shape recovery [65]. The degradation of devices at smaller bending radius was associated with the breakage of perovskite layer. Hu et al. prepared glycol and Zonyl FS-300 modified PEDOT:PSS as the highly conductive transparent electrode which has a low sheet resistance of 40 Ω/sq, and a polystyrene-doped nanocellular PEDOT:PSS HTL in FPSCs to synchronously enhance mechanical flexibility and light utilization. As a result, a PCE of 12.32% was achieved for large-scale FPSCs with excellent flexural endurance, maintaining over 90% of the initial PCE after 1000 cycles with a 2-mm-curvature radius [70].

The conductivity of PEDOT:PSS could be improved by strong acids soaking treatments due to the phase separation and the removal of insulating PSS [68]. But the strong acid is corrosive to polymer substrates. By employing gentle methanesulfonic acid (MSA) to treat PEDOT:PSS film as the transparent electrode in FPSCs, Sun et al. achieved a PCE of 8.6%. They showed that the MSA-PEDO:PSS film with high optical transmittance and good mechanical properties has a great potential in replacing ITO electrode, although it demonstrates much lower conductivity (~ 2,000 S cm^−1^) than ITO on glass (~ 6,000 S cm^−1^) and relatively higher roughness caused by acid treatment. In addition, the MSA modification can lower the work function of PEDOT:PSS from 5.0 to 4.8 eV, which is energetically favorable for carrier transfer and collection [71]. Another approach that can address the negative effect of strong acid on plastic substrates is the acid-assisted transfer-printing strategy by avoiding the direct contact between the acid and plastic substrates (Fig. 2b) [72]. Zhang et al. conducted FPSCs with PDMS transfer-printed PEDOT:PSS as electrodes. Due to the high conductivity of HNO_3_-modified PEDOT:PSS, the devices on PET substrates achieved a PCE of 10.3% and good mechanical flexibility [23].

However, this transfer-printing strategy is not suitable for roll-to-roll manufacture due to the complicated process. And this method lacks reproducibility due to the requirement of precise control of van der Walls interactions at interfaces [68]. At the same time, strong acids usually cause environmental and safety issues. The acid-free approach which combines polar solvent doping and post-treatment provides a better choice in improving the conductivity of PEDOT:PSS, which can be attributed to the rearrangement of PEDOT:PSS and the removal of insulating PSS. The conductivity of PEDOT:PSS has been improved to as high as 5012 S cm^−1^ via this acid-free method with additional oxygen plasma treatment. Moreover, the as-fabricated PEDOT:PSS layer owns a low sheet resistance of 15 Ω/sq and an average optical transmittance of 76%, which is favorable for the application of flexible transparent electrodes [73]. In addition, the ionic additive like Zn(TFSI)_2_ can also effectively enhance the conductivity and flexibility of PEDOT:PSS attributing to the doping effect of CF_3_SO_2_^-^ group in Zn(TFSI)_2_ and the regulated phase separation of conductive PEDOT:PSS network. With the assistance of the fluorosurfactant dopant Zn(TFSI)_2_ and the optimized solution shear stress during slot-die printing, Song et al. prepared highly conductive PEDOT:PSS network electrodes (4100 S cm^−1^) on PET substrates via a roll-to-roll process. The PEDOT:PSS film showed excellent optical clarity and improved flexibility due to the network structure. The FPSCs based on this transparent electrode exhibited stabilized PCE of 19.0% and 10.9% with effective areas of 0.1 and 25 cm^2^, respectively. The corresponding devices also showed excellent stability and outstanding mechanical flexibility, retaining 85% of their initial PCEs after 5,000 bending cycles at a curvature of 3 mm [74]. Later, they improved the efficiency of PEDOT:PSS-based FPSCs to over 20% with an effective area of 1.01 cm^2^ by passivating perovskite grain boundaries [75]. Combining with highly conductive metal gride is also useful in enhancing the electrical performance of PEDOT:PSS, which will be discussed in Sect. 2.4.2.

Apart from the wide application in the bottom electrode, there are few studies using PEDOT:PSS as the top electrode, because the deposition of PEDOT:PSS aqueous solutions is destructive to the underlying perovskite layer. Lately, Song et al. employed the highly conductive PEDOT:PSS on the PDMS substrates as both bottom and top electrode in FPSCs (Fig. 2c), and the top PEDOT:PSS electrode is fabricated by a film-transfer lamination technique. Consequently, the FPSCs demonstrated a PCE of 15.1% with a small area and 7.91% on a large area of 56 cm^2^. Moreover, negligible efficiency decrease was found after 5000 bending cycles at a small curvature radius of 2 mm, and merely 10% decline after 5000 crumpling cycles [19]. In addition, there are still environmental stability problems, including UV light, high temperatures and humidity, which restrict the widespread applications of PEDOT:PSS films.

Section 2.2 has demonstrated that the conductive polymer PEDOT:PSS is a good flexible electrode candidate in FPSCs with the highest PCE over 20% in FPSCs, which is comparable to the ITO counterpart. Compared with brittle ITO electrode, the solution-processability and higher mechanical flexibility make PEDOT:PSS more appealing for application in flexible devices. The main concern in the application of FPSCs is the poor electrical conductivity which can be improved through the doping of polar solvents, acids, and ionic liquids as summarized above. Moreover, the acid-free approach which combines polar solvent doping and post-treatment provides a better choice in improving the conductivity of PEDOT:PSS, which can be attributed to the rearrangement of PEDOT:PSS and the removal of insulating PSS.

### Carbon Nanomaterials

Carbon nanomaterials such as CNTs and graphene are viable alternatives to ITO electrodes in FPSCs owing to their earth-abundant carbon composition, excellent chemical and environmental robustness, ultra-flexibility, and direct roll-to-roll processability [76]. With superior optical and electrical properties, the transparent carbon nanomaterials demonstrate comparable performance to ITO counterparts in FPSCs [77].

#### CNTs

**Fig. 3:**
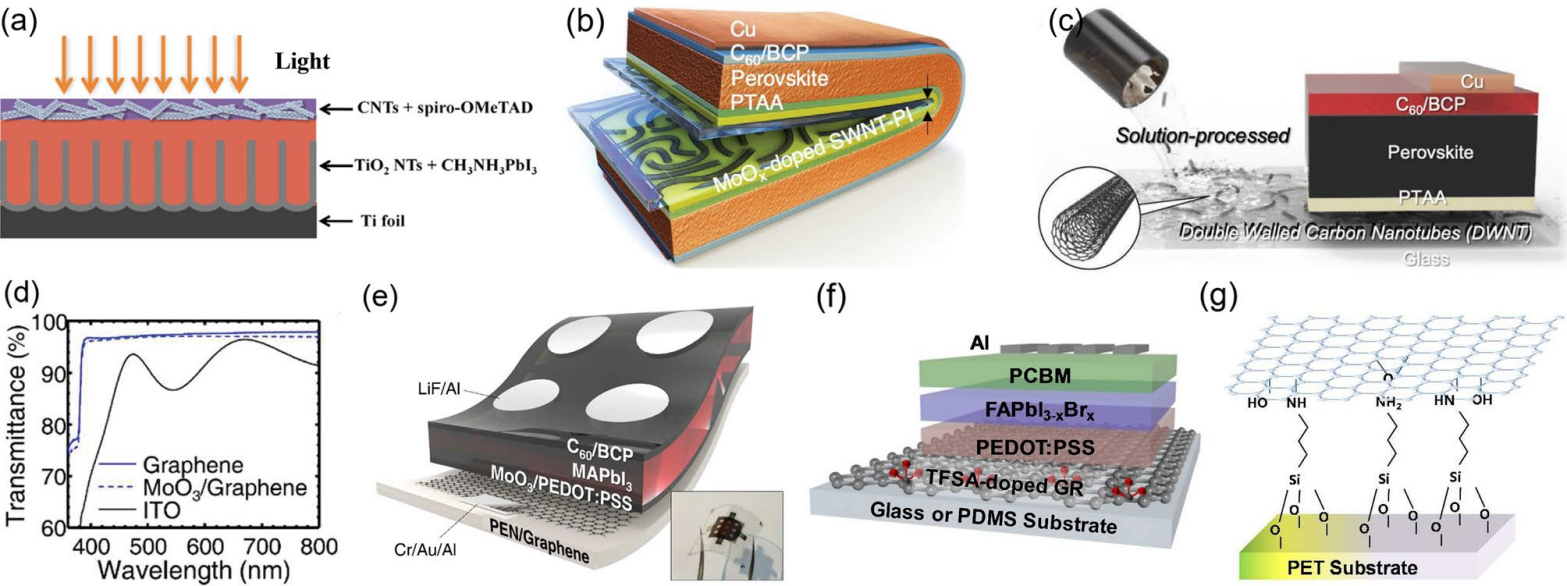
**a** Schematic device structure of FPSCs based on CNTs top electrode. Reproduced with permission from Ref. [78]. Copyright 2015, Elsevier. **b** Foldable PSCs employing SWNTs-embedded PI substrate. Reproduced with permission from Ref. [91]. Licensed under a Creative Commons Attribution (CC BY) license. **c** Schematic illustration of PSC structure using solution-processed DWNTs as transparent electrode. Reproduced with permission from Ref. [92]. Copyright 2019, John Wiley and Sons. **d** Transmittance spectra of graphene/PEN, MoO_3_/graphene/PEN, and ITO/PEN substrates. **e** Device structure of FPSCs based on MoO_3_ doped graphene electrode (The inset image is the picture of full device). **d**, **e** Reproduced with permission from Ref. [96]. Copyright 2017, Royal Society of Chemistry. **f** Device schematic of FPSCs with TFSA doped graphene as electrode. Reproduced with permission from Ref. [77]. Copyright 2018, Royal Society of Chemistry. **g** The covalent bonding between PET and graphene layer with APTES interlayer. Reproduced with permission from Ref. [102]. Copyright 2017, Royal Society of Chemistry

CNTs offer great potential as a low-cost and efficient alternative to expensive and fragile ITO in FPSCs owing to their high-temperature resistance, good optoelectrical performance, excellent air stability, as well as scalable manufacturing capability. Moreover, CNTs possess good reproducibility owing to their facile synthesis and easy transfer strategy. Floating catalyst chemical vapor deposition is a predominant method for CNTs synthesis due to the relatively low production cost, highly efficient and scalable nature [78]. However, due to the van der Waals interactions, CNTs are usually hard to disperse uniformly. And the generally adopted method to deposit CNTs electrode on the substrate is the film transfer strategy with a taped substrate and the aid of several drops of toluene, ethanol or chlorobenzene to ensure firm adhesion and better electrical contact. Laminated aerosol-synthesized CNTs films have been employed on top of PSCs in 2014 [79]. There are many pieces of research that transfer CNTs film on the perovskite layer before depositing the charge transporting layer like CNTs/Spiro-OMeTAD composite electrode (Fig. 3a), which could synergistically facilitate holes extraction and collection [78]. However, these transfer methods are incompatible with large-scale fabrication due to the complex procedures during the device preparation. Furthermore, CNTs can play a wide range of roles except for electrodes in PSCs, such as charge transport layer [80, 81], perovskite additives [82–84] and interlayers [85, 86].

According to the number of concentric graphene cylinders, CNTs could be divided into different types. Single-walled carbon nanotubes (SWNTs) are the simplest class and are mainly composed of a single graphene cylinder [87]. With the advantages of excellent mechanical durability, easy fabrication and high optical transmittance, SWNTs are widely used in PSCs as electrode and hole transporter [79, 88, 89]. The hydrophobic property of SWNTs inhibits moisture invasion, leading to excellent stability for devices. However, this intrinsic hydrophobicity is unfavorable for uniform film fabrication. Additionally, successful doping to improve the optoelectrical properties of SWNTs without undermining cell performance is needed, due to the high sheet resistance and the concession between optical transmittance and conductivity of SWNTs film [88].

Different approaches have been proposed to effectively dope the SWNTs transparent electrodes. Due to the acidic nature, PEDOT:PSS functioned as a mild p-dopant to lower the sheet resistance of SWNTs. But the hydrophilic nature of PEDOT:PSS inhibits the direct deposition to the hydrophobic SWNTs film. Jeon et al. utilized isopropanol modified PEDOT:PSS which is more compatible for application due to the desired wettability on SWNTs film compared to the surfactant modified PEDOT:PSS. However, both modification strategies showed a low shunt resistance and a high sheet resistance in SWNTs electrode, leading to poor performance. Then, they adopted diluted nitric acid (HNO_3_) as an effective dopant, which keeps the equivalent doping effectiveness down to 35 v/v% dilutions. The diluted HNO_3_ can change the surface of SWNTs to hydrophilic to enable the deposition of HTL and enhance the light transmittance of SWNTs films. However, HNO_3_ could damage the structure of SWNTs and the energy level mismatch between HNO_3_-doped SWNTs and perovskite could hinder hole extraction. Nevertheless, they demonstrated FPSCs on the PET/diluted HNO_3_-doped SWNTs substrates and realized a PCE of 5.38% [88]. In comparison with ionic dopants of HNO_3_, the transition metal oxide MoO_3_ is an effective and stable dopant for SWNTs for inducing a strong p-doping effect [90, 91]. However, thicker MoO_3_ with a deep Fermi level results in poor performance due to the energy misalignment between SWNTs and perovskite layer, and the hole transfer is hindered at the interfaces of SWNT/MoO_3_ and perovskite/MoO_3_. Even worse, the thicker MoO_3_-deposited SWNTs film also has problems with poor morphology. With a thin MoO_3_ layer of 2 nm applied on the SWNTs electrode, the doped SWNTs-based FPSCs demonstrated a device performance of 11.0% [90]. Furthermore, CNTs electrodes will peel off under severe mechanical stress due to the relatively low adhesion strength, resulting in poor mechanical durability of CNTs electrodes at a small bending radius. Yoon et al. fabricated a CNT-polymer matrix as depicted in Fig. 3b, which embeds CNTs into polymer films and demonstrates outstanding mechanical robustness and resilience. The corresponding foldable PSCs can keep the initial PCE values after 10, 000 folding cycles with a radius of 0.5 mm [91].

There are also double-walled CNTs (DWNTs) and multi-walled CNTs (MWNTs). Contrary to the hydrophobic SWNTs, the MWNTs are solution-processable. The DWNTs which comprise two concentric graphene cylinders, combine the advantages of MWNTs and SWNTs, exhibit good transparency and conductivity while retaining the solution processability and chemical stability [92]. Recently, Jeon et al. compared the doping behavior between SWNTs and DWNTs and found the doping effect of DWNTs is limited since the inner walls are hard to dope. The optical transmittance of MWNTs film is inferior across the visible region than SWNTs [93]. In addition, the PSCs based on the modified DWNTs (Fig. 3c), which exhibited smoother film morphology and more favorable energy level alignment than SWNTs, achieved a high performance up to 17.2%, indicating the application prospects of DWNTs in flexible devices [92].

#### Graphene

Despite the individual CNT having a high conductivity, the inter-tube contact resistance of CNTs is high, which limits the conductivity of the CNTs film [94]. Compared to CNTs, the single-atomic-thick graphene is smoother, more conductive, and more transparent in a broad wavelength region. For example, the 2-layered graphene/PET electrode demonstrates a ~ 300 Ω/sq sheet resistance and 87.3% optical transmittance at the wavelength of 550 nm [95]. The transmittance of graphene films is even higher than ITO especially in the near-infrared range as demonstrated in Fig. 3d, making graphene an attractive candidate as the transparent electrode in FPSCs [90, 96]. Besides, graphene is chemically and mechanically stable, and usually synthesized on Cu, Ni or Pt foils by chemical vapor deposition (CVD) method and transferred onto substrates by wet-transfer or dry-transfer, although the transfer step for graphene has lower reproducibility [94, 97]. Encouragingly, the polymer/graphene substrates are already available for purchase. Graphene sheets that have been introduced in the FPSCs since 2016 are usually adopted as both bottom and top electrodes [97]. For instance, Heo et al. applied the graphene/PDMS top electrode by lamination with double clip pressure and further thermal process [98]. Moreover, graphene has also been used in PSCs as an electron transport layer (ETL) [99] or HTL [100] besides electrode, which is similar to the multi-role CNTs.

Theoretically, graphene has ultrahigh carrier mobility, but its carrier mobility is limited by the film fabrication quality and generally much lower than the theoretical value. The CVD synthesized single-layer graphene has imperfect morphology with small grain sizes and high-resistance grain boundaries, resulting in high sheet resistance (over 1 kΩ/sq) in the pristine graphene layer [94]. Stacking the graphene sheets and chemical doping are two major methods to improve the conductivity of graphene by reducing the sheet resistance to tens to hundreds of Ω/sq. However, when stacking the graphene sheets, there is a trade-off between the electrical conductivity and the optical transmittance, and the optimal number of graphene layers needs to be controlled [98]. Proper doping is an effective way of impacting both conductivity and Fermi level, and the widely used dopants include MoO_3_, AuCl_3_ and bis(trifluoromethanesulfonyl) amide (TFSA).

MoO_3_ is a favorable p-type dopant for graphene because it enables a desirable energy level alignment by elevating work function, which facilitates the hole extraction from HTL to graphene anode. Moreover, the deposition of MoO_3_ also changes the hydrophobic graphene surface to hydrophilic to ensure the following film fabrication like PEDOT:PSS [96, 101]. With the doping of MoO_3_, Choi and co-workers applied wet-transfer 2D graphene as the transparent electrode and PEDOT:PSS with complete coverage as HTL (Fig. 3e). Although the MoO_3_/graphene still showed a higher sheet resistance of 552 Ω/sq compared to the 13.3 Ω/sq of ITO electrode, the better aligned energy level and higher transmittance made the MoO_3_/graphene-based FPSCs achieve a high PCE of 16.8% with no hysteresis which is comparable to the device based on ITO electrode (17.3%). In addition, the device exhibited outstanding flexibility and retained 85% of the original PCE after 5000 bending cycles at a radius of 2 mm [96]. As stated above, MoO_3_ doping is also employed in SWNTs electrodes. For comparison, Jeon et al. demonstrated the TCO-free inverted FPSCs by replacing ITO with MoO_3_ doped SWNTs or MoO_3_ doped graphene and compared their efficiencies and mechanical resilience. Owing to the better film morphology and higher optical transmittance, the graphene-based FPSCs showed a higher efficiency of 13.3% than the SWNTs-based FPSCs with a PCE of 11.0% [90]. But the SWNT-based FPSCs showed a higher resilience compared to the graphene-based cells owing to the randomly oriented entanglement of SWNTs and low defect sites. Overall, they identified that graphene is a better option as the flexible electrode in FPSCs.

AuCl_3_ could also lead to heavy p-type doping in graphene films, resulting in increased electrical conductivity and controllable work function. Heo et al. used this AuCl_3_ doped single-layer graphene as a transparent electrode on the PET substrates with 3-aminopropyl triethoxysilane (APTES) as interlayer, resulting in a notably declined sheet resistance of graphene (~ 80 Ω/sq). The FPSC based on PET/APTES/AuCl_3_-graphene substrates demonstrated high efficiency of 17.9% with excellent flexural endurance [102]. However, the AuCl_3_ dopant will slightly lower the transmittance of graphene, while the TFSA is an effective dopant that causes no optical loss. The TFSA-doped graphene has high conductivity, excellent transparency, and good stability. By employing the TFSA-doped graphene film as the transparent electrode on the APTES coupled PDMS substrates (Fig. 3f), Heo et al. fabricated highly efficient and stable FPSCs, exhibiting the best PCE of 17.8% for forward and 18.3% for reverse scan direction with good mechanical flexibility, and maintaining 82.2% of the initial PCE after 5000 bending cycles at bending radius of 8 mm [77].

Another challenge about the application of graphene is the weak adhesion between graphene and substrates due to the physical transfer procedure and the absent chemical bonding, resulting in insufficient contact and severe mechanical deformation during flexural experiences [96]. It is necessary to develop more sufficient contacts for graphene to realize ultra-flexible devices. The aforementioned APTES is exactly functioned as the interlayer to improve the adhesion of graphene sheets by forming chemical bonding between graphene and substrates (Fig. 3g), leading to significantly improved mechanical robustness of substrates [77, 102]. Cross-linkable olefin-type polymer interlayer has been also used to improve the adhesion of graphene electrodes and decrease the surface roughness of the substrate. Based on the configuration of PET/olefin-type polymer/graphene/P3HT/perovskite /PCBM/Ag, the FPSCs with an efficiency of 11.5% and good air stability were achieved [97].

Section 2.3 has identified the transparent carbon nanomaterials as promising candidates for the flexible transparent electrodes in FPSCs. Both CNTs and graphene exhibit excellent chemical stability and mechanical flexibility which are beneficial to realize low-cost, highly stable and durable FPSCs. In comparison, CNTs films are more robust during the bending process, while graphene film could provide a much smoother surface to guarantee the quality of the subsequent layer. However, FPSCs based on carbon nanomaterials electrodes usually exhibit lower efficiency than ITO-based FPSCs. This is mainly due to the poor electrical conductivity caused by the high inter-tube contact resistance of CNTs and the imperfect morphology of graphene films. Various dopants used to enhance their electrical conductivity like PEDOT:PSS, acids and MoO_3_ for CNTs and graphene are summarized in this section. And their conductivity can be further increased by combining with other materials, for instance, the incorporation of graphene with AgNWs as described in Sect. 2.4.1. Moreover, to achieve mechanically robust FPSCs using carbon nanomaterials as flexible transparent electrodes, appropriate methods are needed to enhance their adhesion to substrates, including physical embedding electrodes into polymer substrate and introducing interlayer between substrate and electrode.

### Metallic Nanostructures

Graphene, CNTs, and PEDOT:PSS are promising flexible transparent electrodes in FPSCs due to the low-cost preparation process and outstanding optical transmittance in the visible region. Although various modification strategies have been applied to improve their electrical properties, their conductivities are inferior to metallic electrodes. Considering the excellent electrical conductivity and broad optical transparency, metallic nanostructures including metal nanowires, UTMFs and metal meshes are strong candidates to replace ITO electrodes in FPSCs. In Sect. 2.4, we review metallic nanostructured electrodes and focus on key issues and improvements for their applications in FPSCs.

#### Metal Nanowires

Metal nanowires, especially silver nanowires (AgNWs) are considered the most promising metal nanowires owing to their cost-effective fabrication techniques and outstanding optoelectrical properties. The highly flexible AgNWs transparent electrodes could enable the roll-to-roll fabrication processes and its low sheet resistance at a transmittance of 80–90% is comparable to ITO. Besides, the high haze ratio caused by light scattering from AgNWs facilitates the light harvest of solar cells [103]. As for the film fabrication, AgNWs can be easily deposited by conventional solution-based processes, such as spin coating [104], vacuum filtration and roll-to-roll compatible techniques, such as spray coating [105–107], bar coating and slot-die coating [108] which are favorable in realizing large-scale fabrication process.

The critical issues of AgNWs include poor chemical stability, high surface roughness, low coverage (less than 40%), high junction resistance, and low adhesion of AgNWs on the polymer substrates [109]. The strong reaction of the silver and halides results in the formation of insulating silver halide, causing a sharp increase of sheet resistance and the degradation of AgNWs-based transparent electrode [108, 110]. In addition, corrosion and oxidation can easily happen when AgNWs network is exposed to air or harsh environments. The rough surface caused by the agglomeration of nanowires during the solution process leads to a short circuit and corruption of perovskite and AgNWs electrode. Various ways have been reported to gain a smooth surface, such as depositing barrier layers (transparent metal oxides [111–115], PEDOT:PSS [108, 116], graphene [104, 110]), or using special deposition methods like capillary printing [8], which will be discussed in detail in the following paragraphs.

**Fig. 4:**
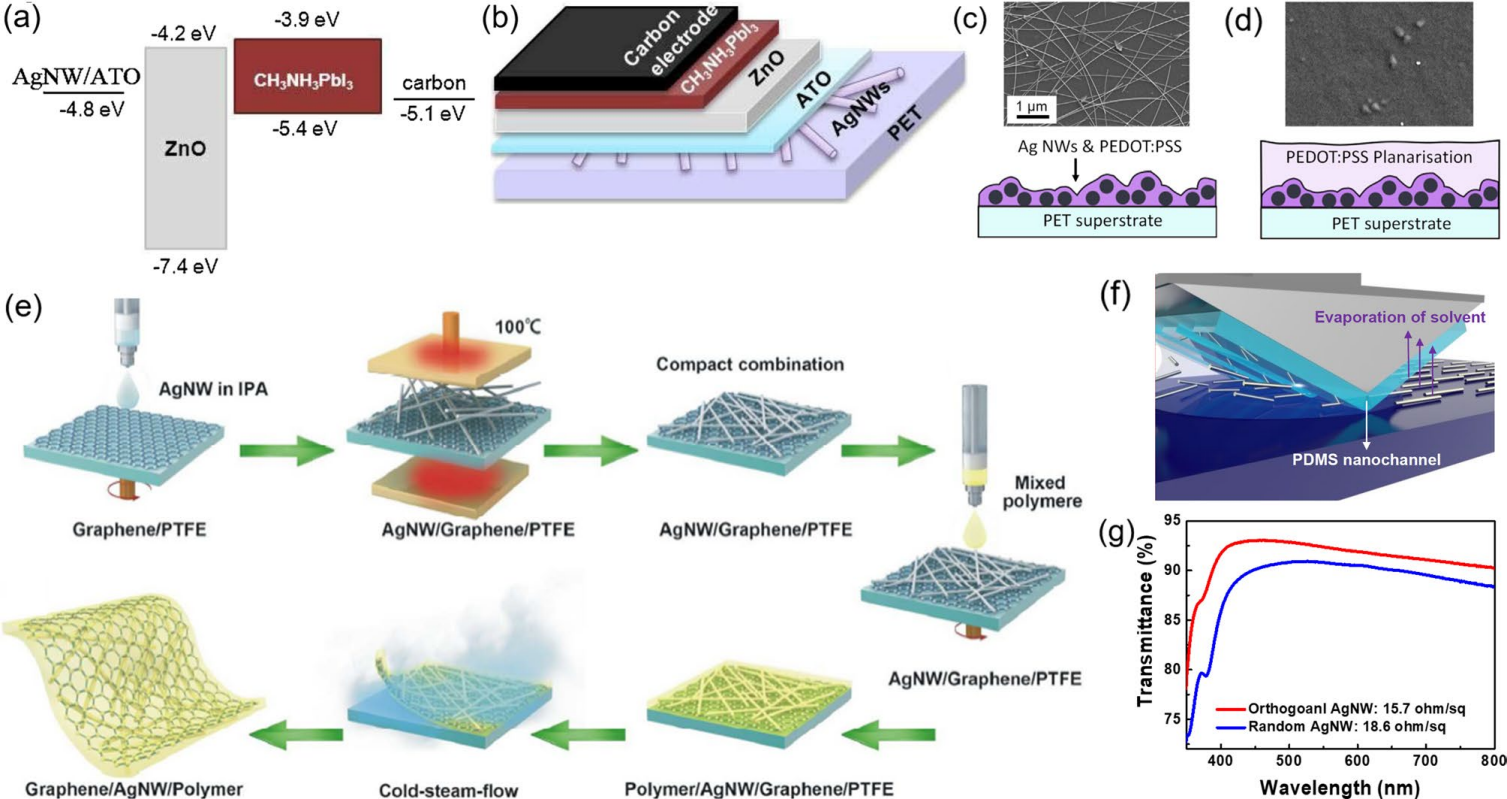
**a** Energy level diagram and **b** device structure of FPSCs based on PET/AgNWs/ATO substrate and carbon back electrode. Reproduced with permission from Ref. [41]. Copyright 2020, Elsevier. SEM images and schematic diagrams of different AgNWs/PEDOT:PSS composite electrodes **c** without and **d** with planarization layer. Reproduced with permission from Ref. [108]. Copyright 2017, John Wiley and Sons. **e** Schematic illustration of the procedures in fabricating composite PET/graphene/AgNWs electrode. Reproduced with permission from Ref. [110]. Copyright 2016, American Chemical Society. **f** Schematic of fabricating orthogonal AgNWs via capillary printing and **g** the optical transmittance of orthogonal AgNWs/glass compared with random AgNWs/glass. **f** Reproduced with permission from Ref. [118]. Copyright 2015, American Chemical Society. **g** Reproduced with permission from Ref. [8]. Copyright 2019, Royal Society of Chemistry

In addition to providing a smooth surface, the protective barrier should have good ion impermeability, good mechanical robustness, and high optical transparency to minimize the additional energy loss. TCO materials, such as ITO, AZO and ZnO, have been inserted between the metal electrode and the halide perovskite as a protective layer. Cao et al. deposited fluorine doped ZnO (FZO) above AgNWs layer, which demonstrated improved conductivity, chemical and mechanical stability [114]. Im et al. protected the AgNWs electrode by depositing a 10 nm thick ITO layer in vacuum, and the resulting flexible devices show high efficiency of over 14% with excellent chemical stability [113]. Lately, Jon et al. introduced chemical robust and cheap ATO onto AgNWs film (Fig. 4a–b). This composite transparent electrode of AgNWs/ATO showed a transmission of 76–82% in the visible region with a low sheet resistance of 18 Ω/sq, similar to the commercial ITO/PET transparent electrodes [41]. In the AgNWs-based composite electrode, a moderated conductivity is enough for the encapsulation materials since the role of encapsulation material is mainly for protecting AgNWs from deterioration, while the electrical conductivity mainly arises through the metal nanowire networks. Therefore, highly transparent ZnO which can be processed by the low-temperature solution methods has also been adopted. The ZnO interlayer provides a smoother surface and isolates the AgNWs layer with perovskite to restrain Ag diffusion while having a slight influence on light transmittance and sheet resistance. Jin et al. introduced a sol-gel-derived ZnO barrier layer on AgNWs film and further deposited a compact and pinhole-free TiO_2_ film to inhibit the decomposition at the ZnO/perovskite interface. The FPSCs based on PET/AgNWs/ZnO/TiO_2_ electrode demonstrated a best PCE of 17.11%, which is comparable to the device on ITO/glass electrode (18.26%) [112]. However, as mentioned above, the insufficient flexibility of TCO materials limits its application in flexible devices.

Conducting polymer PEDOT:PSS with high mechanical flexibility is favorable as the interlayer between AgNWs and perovskite. Incorporation of PEDOT:PSS and AgNWs has advantages in improving surface flatness, adhesion to substrates and electrical conductivity by filling the uncovered area and connecting the silver nanowire junctions [116]. Vak and co-workers fabricated AgNWs/PEDOT:PSS transparent electrodes upon PET substrates via a scalable, roll-to-roll slot-die process. Specifically, AgNWs were dispersed in PEDOT:PSS and surfactants, and then slot-die coated in a roll-to-roll setup. After that, the planarization layer PEDOT:PSS was coated in the same method which promoted the formation of uniform perovskite film and functioned as both HTL and barrier layer (Fig. 4c–d). Based on this transparent electrode, they realized a PCE of 11% in TCO-free FPSCs with excellent mechanical flexibility. Negligible change in efficiency was found after 10,000 compressive bends at a 5 mm radius [108]. Recently, a composite electrode PH1000/AgNWs fabricating on the transparent biomass polylactic acid substrates was reported, exhibiting an average transmittance of more than 82% in the wavelength region of 400–800 nm with a low sheet resistance of 25 Ω/sq. Furthermore, the FPSCs based on this composite electrode demonstrated a fair PCE of 11.44% with good flexibility [116]. However, the parasitic optical absorption and the acidity of PEDOT:PSS would hamper the light harvest and stability of devices. Moreover, there is a trade-off between the transmittance and conductivity of the electrodes, that is, a thinner PEDOT:PSS film will increase the transmittance of composite electrodes coming at the expense of lower conductivity [108, 117].

Due to the high chemical and thermal stability, high optical transparency, and simple preparation process, combining with graphene is one of the most effective strategies to protect AgNWs. In addition, the graphene film is also capable of providing extra electron pathways for the composite structure. A self-assembled graphene oxide flake was introduced by Lu et al. as the anti-corrosive barrier on AgNWs in FPSCs. They demonstrated that the amount of graphene oxide flakes is vital to the formation of a flawless barrier while maintaining high electrical conductance. Moreover, the partially reduced graphene oxide flakes by NaBH_4_ demonstrate good energy level alignment and surface wetting property in the application of FPSCs, resulting in a PCE of 7.92% [104]. Dong et al. encapsulated the AgNWs with a sandwich structure between the monolayer graphene and the polymer matrix (mixture of polycarbonate, polystyrene and additives) to resist degradation and the fabrication procedures are presented in Fig. 4e. This hybrid electrode exhibited a sheet resistance as low as 8.06 Ω/sq and a light transmittance of 88.3% at 550 nm. With the merits of low surface roughness and high mechanical toughness, they demonstrated a MAPbI_3_-based FPSC with an efficiency of 10.4% [110].

However, these methods with additional protection layers are incompatible with large-scale solution processing due to the complicated fabrication process. Additionally, the random AgNWs network fabricated by conventional solution methods has low optical transmittance and high surface roughness, causing an inferior device performance. Recently, Kang et al. employed orthogonal AgNWs transparent electrodes on the 1.3 μm thick PEN substrate via capillary printing process. In this method, the nanowires were aligned by the meniscus surface tensions which were formed between PDMS nanochannels and substrates, leading to smoother surface morphology and higher optical transmittance (92.3% at 550 nm) compared to random AgNWs (90.8% at 550 nm) as demonstrated in Fig. 4f–g. The direct contact between the even orthogonal AgNWs electrode and the perovskite film was effectively prevented. Consequently, the formation of the silver halide is prohibited, resulting in no electrical conductivity loss during the entire fabrication process. With the passivation layer PH1000 and HTL AI4083, the orthogonal AgNWs electrodes-based FPSCs achieved a PCE of 12.85% with a high power-per-weight of 29.4 W g^−1^, which proved the orthogonal AgNWs network is an effective strategy to address the challenges in the application of AgNWs [8, 118].

**Fig. 5:**
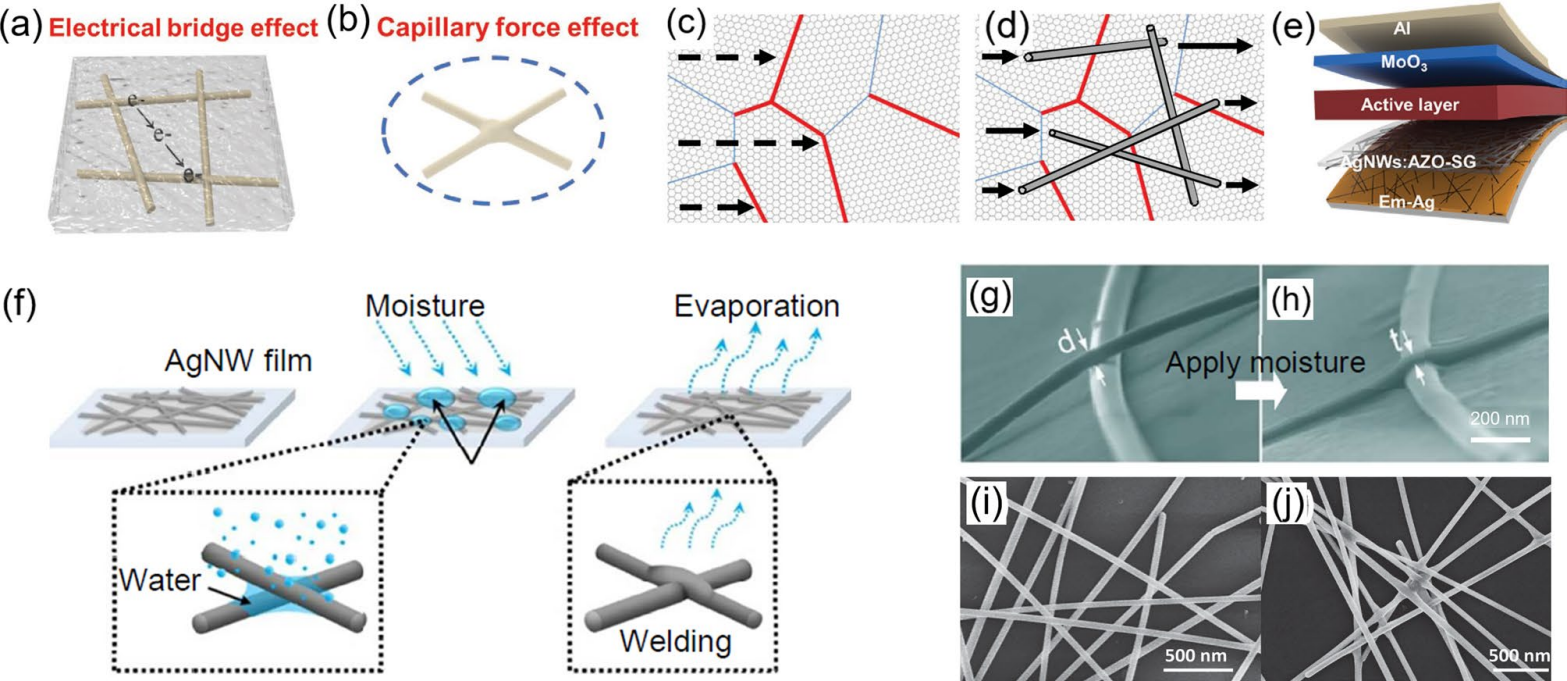
Illustration of **a** the electrical bridge effect and **b** the capillary force effect. The illustration of the co-percolating conduction for AgNWs/graphene composite electrode (**d)** compared to the single layer graphene with has high-resistance grain boundaries (red lines) (**c)**. **e** Schematic illustration of FPSCs based on the embedded-Ag/AgNWs: AZO-graphene hybrid electrode. **f** Schematic of welding the AgNWs via water treatment induced capillary force. The SEM images of AgNWs junctions **g** before and **h** after water treatment, **i** before and **j** after chemical treatment. **a**, **b**, **e** Reproduced with permission from Ref. [109]. Copyright 2020, John Wiley and Sons. **c**, **d** Reproduced with permission from Ref. [120]. Copyright 2013, John Wiley and Sons. **f, g, h** Reproduced with permission from Ref. [122]. Copyright 2017, American Chemical Society. **i, j** Reproduced with permission from Ref. [124]. Copyright 2015, John Wiley and Sons

AgNWs have high conductivity in nanowires, but high sheet resistance at the AgNWs junction sites. This large contact resistance between nanowires would generate a lot of heat and limit the conductivity of AgNWs films, inevitably inhibiting their application in FPSCs. The conventional method to reduce the high junction resistance of AgNWs is welding. A variety of techniques have been developed such as the post-treatment with the assistance of heating [41, 110], electricity [119], mechanical pressure or light, while all have concerns in the application of flexible devices. Besides, coating with additional materials on AgNWs films including PEDOT:PSS [108, 116], graphene [104, 110], AZO [109] and so on is effective due to the electrical bridge effect and the capillary force effect (Fig. 5a–b). A composite electrode has been reported by Chen et al. in 2013, which combines the AgNWs network with single-layer graphene. Owing to the co-percolating conduction, nanowires and the high-resistance grain boundaries in graphene are bridged by each other to reduce the contact resistance as illustrated in Fig. 5c–d. This hybrid electrode achieved superior photoelectrical characteristics with a low sheet resistance of 22 Ω/sq and high optical transparency of 88% at 550 nm wavelength [120]. Additionally, the problem of AgNWs peeling from substrate during bending cycles will drop the current density and decrease the efficiency of flexible devices [121]. Recently, Li et al. proposed a flexible transparent electrode by combining the upper electrode and the underlying AgNWs-embedded PET substrates (Fig. 5e). The upper electrode comprised AgNWs network which is welded by the capillary force effect and the secondary growth of AZO, resulting in smooth surface, excellent photoelectrical properties and enhanced mechanical peeling stability [109]. However, coating additional materials would alter the work function of AgNWs electrodes. Apart from that, the post-treatment with water or moisture is promising in welding the nanowires due to the water evaporation during the drying process, which will induce a strong capillary force effect to weld the junctions as shown in Fig. 5f (schematic diagram), g-h (SEM images) [122]. Hu et al. realized the AgNWs electrode with high electrical properties by using the rapid radial electrochemical etching to tailor the diameter of AgNWs and post-treatment with water [123]. Besides, the localized chemical reaction of AgNWs also works, which involves the reduction of Ag atoms particularly at the nanowires contact sites owing to the capillary force (Fig. 5i–j), resulting in an increased contact area and an effectively decreased sheet resistance of 20.5 Ω/sq [124].

When AgNWs are employed as the top electrode, it is favorable for preparing semi-transparent PSCs. However, the solvent of solution-processed AgNWs may damage the underlying layers in PSCs. The spray-coating method with low boiling point solvent like IPA which has an ultrafast drying process is a feasible strategy in fabricating AgNWs top electrode [105, 107]. In contrast, the blade coating and spin coating could result in a fast breakdown of the perovskite layer [106]. Besides, some studies adopt rational interface engineering to protect the underlying layers by inserting ZnO interlayer under the AgNWs top electrode [106, 125]. Furthermore, AgNWs are prone to oxidation and corrosion when exposed to ambient conditions. Thus, reasonable encapsulation methods for AgNWs-based devices are required to improve device stability [125].

#### Metal Meshes

**Fig. 6:**
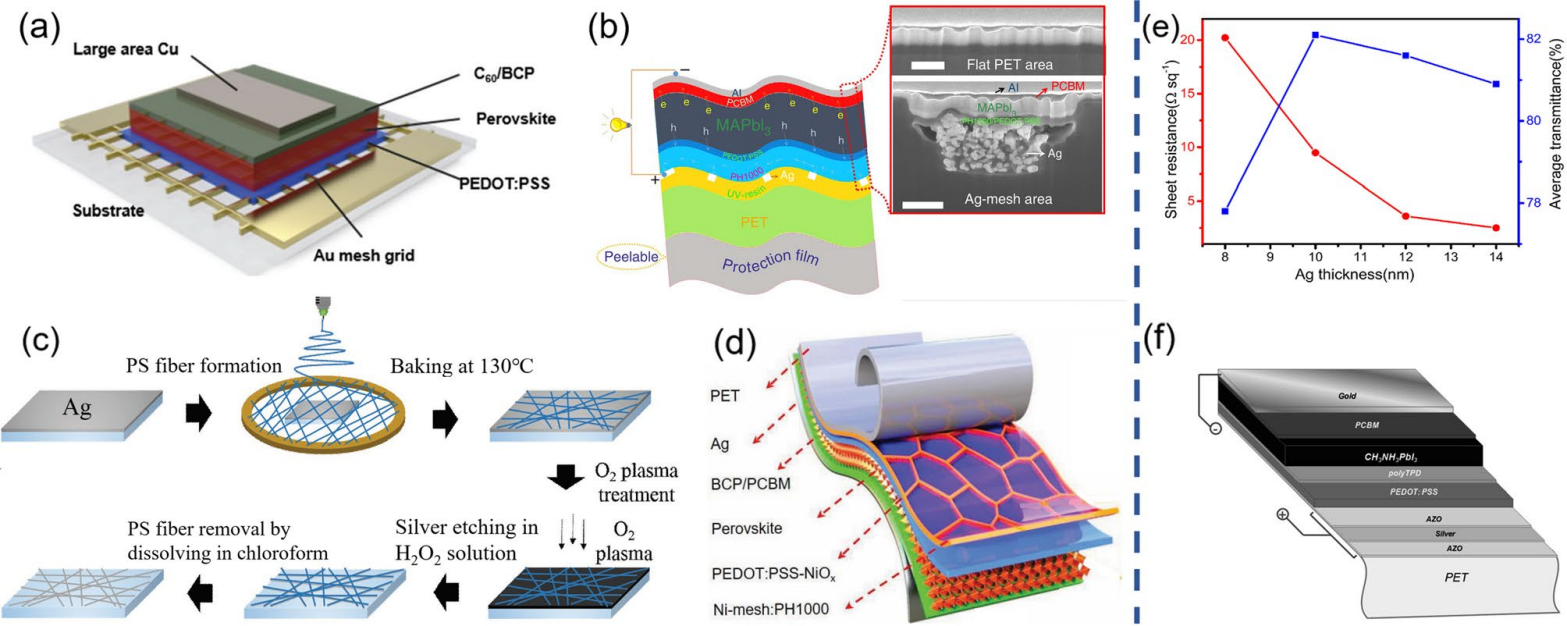
**a** Schematic layout of FPSCs based on Au mesh/PEDOT:PSS electrodes. Reproduced with permission from Ref. [22]. Copyright 2019, Royal Society of Chemistry. **b** Diagram of the FPSC based on the Ag-mesh/PH1000 electrode and the corresponding cross-section SEM image. Reproduced with permission from Ref. [126]. Licensed under a Creative Commons Attribution (CC BY) license. **c** Schematic of fabricating Ag network electrode. Reproduced with permission from Ref. [131]. Copyright 2017, John Wiley and Sons. **d** Schematic illustration of FPSCs based on PET/Ni-mesh/PH1000 electrode. Reproduced with permission from Ref. [134]. Licensed under a Creative Commons Attribution (CC BY) license. **e** The dependence of sheet resistance and average transmittance to Ag thickness in OMO structure electrode. Reproduced with permission from Ref. [137]. Copyright 2019, Elsevier. **f** Structure of FPSCs based on the AZO/Ag/AZO electrode. Reproduced with permission from Ref. [139]. Copyright 2014, Royal Society of Chemistry

Due to the outstanding electrical conductivity, good light transmittance and mechanical flexibility, metal meshes are promising candidates to compete with the massively used TCO electrode, especially in flexible devices. Metal meshes including Ag, Au, Cu and Ni mesh are usually combined with other transparent conductive materials to form highly efficient composite electrodes. Ag mesh/PEDOT:PSS composites have been successfully developed to replace ITO/PEN transparent electrodes in flexible PSCs due to their excellent optical and electrical performance [126]. However, the device stability is impeded due to the electrochemical corrosion between the Ag electrode and perovskite materials, which is found that the reduction of PH1000 and oxidation of Ag are the major causes of this corrosive reaction. Zhang et al. reported that the modified PH1000 with ammonia and polyethylenimine could serve as a barrier above Ag grid electrode to effectively suppress this redox reaction, since ammonia could adjust the pH and the polyethylenimine could reduce PH1000 prior to Ag. As a result, they improved the PCE of corresponding devices from 3.13 to 14.52% [127]. Some researches demonstrated Au mesh electrodes. Jin et al. prepared Cr (3 nm)/Au (60 nm) grid via photolithography and etching processes on the NOA63/CPI substrates. Subsequently, PEDOT:PSS with EG modification was coated above to form a hybrid transparent electrode. This electrode demonstrated excellent mechanical durability for 10,000 bending cycles at a radius of 0.7 mm and the resultant devices showed a PCE of 12.7% [128]. Choi et al. demonstrated large area FPSCs on Au mesh/PEDOT:PSS composite electrodes via photolithography and achieved an efficiency of 13.6% within an area of 1 cm^2^ (Fig. 6a) [22]. Compare to Au and Ag, Cu-based electrodes are cheaper and demonstrate lower diffusion into perovskite film. Recently, Zheng et al. fabricated Cu grids via the photolithography process upon the PET substrates and formed a hybrid electrode with the PH1000 layer. With a Cu top electrode, the resultant FPSCs demonstrated a PCE of 13.58%, retaining over 90% of the original efficiency after 1000 bending cycles at a radius of 5 mm [129].

Metal meshes fabricated by photolithography strategy are costly and time-consuming, which mainly involves evaporating metal films on the pre-patterned photoresist film that has been selectively exposed with a photomask and then stripping the photoresist [22, 128, 129]. There are other more efficient methods in fabricating metal meshes. Yang et al. efficiently fabricated hexagonal Ag mesh by roll-to-roll nano-imprinting lithography [126]. The first step of nano-imprinting is to imprint grooves on a substrate through a patterned mold. Then the grooves are filled with metal nanoparticle ink, and the excess ink is removed by scraping several times. With this strategy, they fabricated highly flexible and ultrathin hybrid electrodes, PET/Ag-mesh/PH1000 as shown in Fig. 6b, which demonstrated a transmittance of 82–86% with a low sheet resistance of 3 Ω/sq, and the corresponding FPSCs achieved efficiency of 14% [126]. In the nano-imprinting strategy, the top-down approach results in low utilization of Ag ink and insufficient conductivity due to pinholes among the metal nanoparticles. Novel fabrication method like bubble-assisted assembling is bottom-up pattern strategy, in which the bubbles obtain patterns with pre-designed shapes and sizes with the help of micropatterned silicon pillars. With this strategy that enables efficient utilization of Ag ink, Yang et al. fabricated high-resolution Ag grids with dense particle stacking. The PET/PDMS/Ag grids demonstrated a low sheet resistance of 7.9 Ω/sq and average light transmittance of over 85% (from 395 to 900 nm), and the corresponding FPSCs exhibited a high PCE of 18.49% [130]. Moreover, there have been studies to fabricate silver networks by dry etching Ag films using randomly distributed electrospinning-prepared polymer nanofiber networks to act as shadow masks (Fig. 6c) [131, 132]. The corresponding junction-free silver nanonetworks demonstrate a high transmittance of 94.4% and a low sheet resistance of 2.4 Ω/sq, showing promising prospects in the application of FPSCs.

Nevertheless, the device degradation caused by the slow migration of metal ions and halide ions would hamper the performance and stability of device. The insertion of a protective layer between the metal electrode and the halide perovskite is a common and effective strategy to inhibit this interdiffusion behavior. The protective barrier should possess good impermeability of ions and high optical transparency. Compared to the brittle TCO, graphene is a suitable and effective barrier layer to prevent ions diffusion in the application of flexible devices. Park et al. deposited hexagonal Cu grids by the electron beam evaporation on the photolithographic patterned SiO_2_ substrates. After the Cu grid embedded in polyimide (PI) was detached from the SiO_2_ substrate, the graphene monolayer was transferred to the top to form a flexible composite electrode. Besides, the graphene layer displays extra charge conducting pathways to further decrease the sheet resistance of the electrode, resulting in outstanding optoelectrical and mechanical characteristics of the composite electrode. And the corresponding FPSCs exhibit excellent chemical stability and approaching performance with glass/ITO counterparts [133]. The Ni-mesh-based electrode is more stable due to the easily formed dense nickel oxide on the surface of Ni mesh to impede further decomposition. Wang et al. prepared the transparent bottom electrode with a configuration of PET/Ni-mesh/PH1000 (Fig. 6d) which shows excellent optical transmittance of about 85–87% and low sheet resistance of about 0.2–0.5 Ω/sq, which is superior to Ag-mesh counterpart. The corresponding FPSCs demonstrated high efficiency of 17.3% with good mechanical durability [134].

However, the formation of a high-quality perovskite film was limited on the metal mesh bottom electrode. Specifically speaking, the conversion of perovskite precursor is spatially non-homogeneous, considering the higher thermal conductivity of Ag-mesh than polymer [96, 126]. When the transparent metal mesh electrodes are applied as top electrode, the non-uniform thermal conductivity is no longer a problem in application. However, there are concerns in preparing metal mesh, since the fabrication processes usually damage the underlying functional layers. Laminating prefabricated metal mesh electrode could solve this problem. By laminating Ni mesh-embedded PET electrode above the device with slight finger pressure, Throughton et al. applied the transparent Ni mesh as the top electrode and realized a PCE of 10.3% [135].

#### Ultrathin Metal Films

Owing to the easy preparation process via low-temperature vacuum deposition and the excellent electrical and mechanical properties, UTMFs with a thickness of approximately 10 nm are promising transparent electrodes. The UTMFs electrodes demonstrate high bending stability under a small bending radius of 1 mm and even foldability attributing to the ductile metal film, which favors the application in flexible devices. Moreover, the high roughness of flexible substrates could be polished by depositing thin metal films due to the sputtering process, resulting in guaranteed quality of perovskite layer [136]. The two essential requirements of UTMFs electrodes (like Au, Ag and Cu) are the high optical transmission for sufficient light utilization and high electrical conductivity for effective charge collection. Both highly relied on the film thickness of metal electrodes. Generally, when the thickness of metal film exceeds the threshold value to transfer island-like film to continuous film, there is a trade-off between the optical transmittance and the electrical conductance as illustrated in Fig. 6e [137]. Thus, the optimization of film thickness is needed to maximize device efficiency. Besides, the nucleation and the growth kinetics of metal films control the film continuity which is related to the electrical conductivity of metal film. The additional seed layer and modification layer are favorable for the growth of UTMFs. Cu or metal oxide is usually selected as the seed layer to lower the threshold thickness and induce high-quality thin metal films. Gaspera et al. demonstrated that an ultrathin MoO_3_ seed layer (1–2 nm in thickness) can help to obtain a continuous and smooth Au layer (10 nm) with a significant reduction in sheet resistance from ~ 250 to ~ 13 Ω/sq [138]. In addition, MoO_3_, TiO_x_ with high refractive index and low extinction coefficient are deposited on UTMFs, which are favorable as an optical spacer layer to diminish the reflection of metal film. Moreover, the top oxide film also plays the role of a protective layer to prevent chemical reactions with perovskite or oxidation in the ambient. Therefore, the optimized film thickness and the inserted layer are vital in acquiring UTMFs with suitable characteristics for the application of flexible electrode. Especially, when the seed layer and the protective layer are made of the same metal oxide, this alternating structure is also called an oxide/metal/oxide (OMO) electrode.

UTMFs electrodes could be employed as a transparent bottom electrode on polymer substrates via magnetron sputtering method or thermal evaporation. For example, Roladan-Carmona et al. fabricated stacked electrode of AZO (30 nm)/Ag (9 nm)/AZO (30 nm) (see Fig. 6f), which has an optical transparency of 81% in the visible region and sheet resistance of 7.5 Ω/sq [139]. TiO_2_/Ag/TiO_2_ electrode fabricated by Song et al. demonstrates average transmittance of 79.6% with a low sheet resistance of 3.6 Ω/sq. The resulting ultra-flexible devices could maintain 84% of the original PCE after 50 single folding cycles [136]. Except for vacuum deposition methods, solution-based fabrication methods are more conducive to the mass production of UTMFs. Zhang et al. developed a versatile solution process for the preparation of ultrathin Au film, which involves a displacement–diffusion-etch process at mild temperature (< 150 ℃). The as-prepared ultrathin Au film demonstrates a moderate light transmittance of 75% at the wavelength of 550 nm and an average sheet resistance of 45 Ω/sq, indicating promising applications in FPSCs [140]. The prolonged thermal treatment for the solution-processed perovskite film is harmful to the UTMFs, which restrains the application of UTMFs as the bottom electrode. Thus, it is advisable to adopt other deposition methods which do not influence on UTMFs and flexible substrates. By avoiding the annealing treatment, Feng et al. applied vapor deposition technology to prepare perovskite films on the UTMFs-based substrates. The corresponding Au film (7 nm) anode demonstrated a low sheet resistance of 19 Ω/sq with a moderate light transmittance (about 70%) attributed to a pre-deposited 3 nm MoO_3_ seed layer. Resultantly, a PCE of 9.05% with good mechanical flexibility was realized for flexible devices, remaining 74% of the original efficiency after 2000 times bending at a small radius of 3.5 mm [76].

UTMFs have also been employed as a transparent top electrode for the FPSCs based on an opaque substrate or realizing semitransparent FPSCs, and the thickness optimization of metal films is still needed to balance the light transmittance and conductivity [141, 142]. For example, Lee et al. introduced a 12 nm thick Ag film as the top electrode via thermal evaporation [141]. Lately, Hanmandlu et al. introduced a microcavity-structured Ag film cooperating with MoO_3_ as an anode on the PET substrates, and an ultrathin stack layer of Cu (1 nm)/Ag (10 nm)/MoO_3_ (40 nm) as a transparent top cathode. The light path length in the perovskite layer was extended due to the light interference effects that emerged between metal electrodes [143].

Section 2.4 has reviewed the electrode properties and applications of metallic nanostructured materials, including metal nanowires, metal meshes and UTMF. AgNWs film is an appealing candidate as a flexible transparent electrode due to the low cost and solution processability. The key issues of applying AgNWs as transparent electrodes have been summarized in this section, including poor chemical stability with perovskite materials, rough surface, and high junction resistance. These weaknesses of AgNWs electrode could be improved by coating additional materials or employing novel fabrication processes, so efficient and stable FPSCs could be obtained. Metal meshes such as Au, Ag, Cu and Ni meshes are also attractive candidates to replace ITO electrode. Similar to AgNWs film, additional protective layers are required to modify the performance of FPSCs based on metal meshes. Various protective layers such as TCO, conducting polymer and carbon materials are reviewed in this section. Moreover, compared to the solution-processable AgNWs electrode, metal mesh electrodes are usually fabricated by expensive or time-consuming techniques, such as photolithography, nano-imprinting and template methods. New fabrication strategies which allow the scalable manufacturing of FPSCs with metal mesh electrodes are needed to be developed. Finally, we reviewed the stacked structure and thickness control of UTMFs. However, the application of UTMFs electrode in FPSCs is still limited due to the inferior performance which is mainly ascribed to the low light transmittance.

## Flexible Opaque Electrodes

### Metal Films

When the thickness increases to 60–100 nm, metal films turn opaque and highly conductive, and they are the most popular top electrode in FPSCs [50, 126, 144]. The commonly used metal films, such as Au (work function is 5.1 eV), Ag (work function is 4.6 eV), Cu (work function is 4.7 eV) and Al (work function is 4.3 eV) films, are generally fabricated by thermal evaporation [91, 126, 129, 145–148]. The homogeneous and dense morphology of metal film facilitates efficient contact with charge transporting layer [149]. Moreover, the reflection of the metal top electrode could improve the light utilization efficiency of the device. However, the intrinsic and environmental stability of metal film electrodes is worrying, because the metallic bonds are easily broken by iodide from the perovskite layer [150–152]. The perovskite decomposition products hydroiodic acid (HI) which could react with Ag and Al, and the consumption of HI will further drive the decomposition reaction toward the right side (Eq. 1) [152, 153]:

1 $$CH_{3} NH_{3} PbI_{3} \leftrightarrow CH_{3} NH_{2} + HI + PbI_{2}$$

**Fig. 7:**
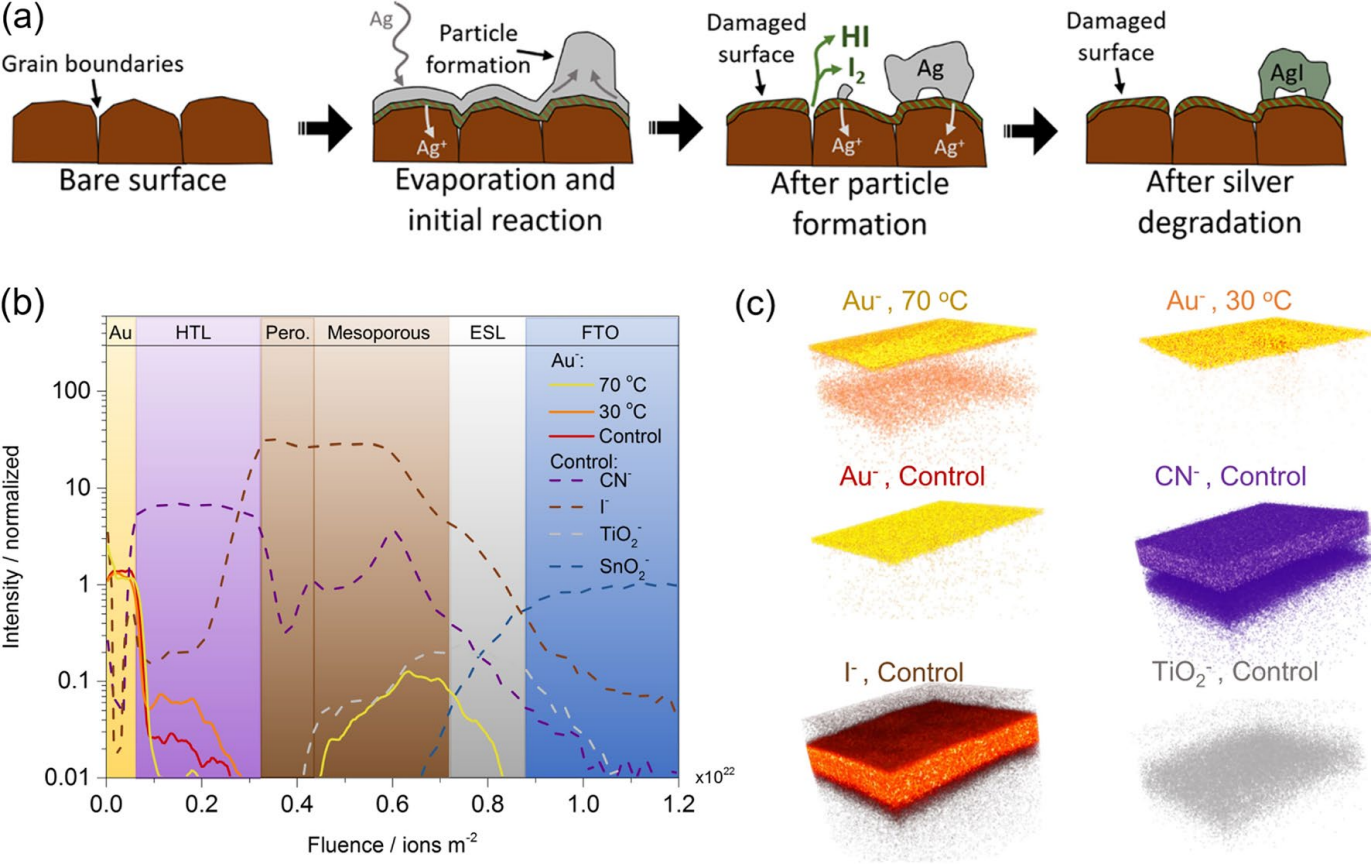
**a** Degradation model of evaporated Ag with perovskite. Reproduced with permission from Ref. [151]. Copyright 2020, American Chemical Society. https://pubs.acs.org/doi/10.1021/acsami.9b20315, please note that further permission related to the material excerpted should be directed to the ACS. **b** Time of flight secondary ion mass spectroscopy elemental depth profiles of the control device and aged devices at 30 and 70 °C. **c** Reconstructed elemental 3D maps for the ions traced in the depth profile. **b**, **c** Reproduced with permission from Ref. [154]. Copyright 2016, American Chemical Society

As illustrated in Fig. 7a, researchers have revealed the reaction mechanisms of thermally evaporated Ag on perovskite film. The formed silver halide barrier and the deteriorated perovskite film that resulted from ions migration leads to serious device degradation [150, 151]. Au is more chemically inert than Ag and Al. However, the long-term stability of devices based on Au electrode is still not satisfying. Grätzel et al. reported that the diffusion of Au film into the perovskite layer under 70 ℃ would cause irreversible performance loss, as shown in Fig. 7b–c [154]. The insertion of a thin chromium barrier layer between HTL and Au could improve the long-term stability of PSCs. Kaltenbrunner et al. introduced Cr_2_O_3_/Cr thin layers between the metal top electrode and charge transporting layer [7]. However, the barrier performance of the buffer layer could not last long for years of operation. Under inert atmosphere, Cu top electrode is stable even in direct contact with perovskite under thermal annealing or light illumination. However, Cu can be oxidized under ambient conditions, and the possible products can react with the perovskite decomposition products, which would further accelerate the deterioration of perovskite [153]. Complete encapsulation is helpful to obtain stable Cu-based FPSCs by eliminating the presence of oxygen and moisture. Besides, an additional capping layer like Al film could also protect the Cu electrode from any adverse environmental impact [129].

### Opaque Carbon Materials

Carbon materials are cheap, chemically stable, inert to ions migration and water-resistant, making them ideal materials to substitute metal films as opaque top electrode. According to the previous research, flexible devices with all carbon electrodes demonstrate superior stability, retaining about 90% of their original efficiency under continuous light illumination or thermal annealing at 60 ℃ in air. In contrast, the flexible devices with Au film electrodes remained 75% after light soaking and 52% after thermal treatment in air, while the devices based on Ag film electrodes were even worse [95]. Due to the capability of extracting photo-generated holes, carbon electrode allows researchers to remove the expensive and unstable HTL to fabricate HTL-free devices, which means an easier fabrication process and better device stability [155]. Carbon black/graphite has been first employed as the top electrode. However, the high-temperature sintering (*e.g.,* 400 ℃) for high conductivity inhibits the application on the flexible polymer substrate [155]. The free-standing carbon films like cross-stacking CNTs could be transferred to flexible device without further thermal treatment [95, 156]. Besides, the printable carbon paste with low drying temperature (100 ℃) and flexible mechanical properties is favorable for flexible devices. The carbon pastes usually contain carbon black, graphite, polymer and additives. And the carbon paste electrode can be easily fabricated by doctor blade coating, painting or screen printing the commercial carbon pastes on perovskite layer, which is favorable for large-scale fabrication [41, 157]. However, the poor contact at the carbon/perovskite interface leads to high series resistance and significant charge recombination. The energy level mismatch between perovskite and carbon electrode limits charge transportation and the device efficiency lags behind those with metal film electrodes. Inserting an interlayer like poly (ethylene oxide) could modulate the energy level alignment at the perovskite/carbon interface [158]. Babu et al. introduced ultrathin chromium (Cr) layer before coating the carbon paste electrode to get good electrical contact at the interface. The corresponding FPSCs with efficient charge collection realized a PCE of 15.18% with a large area of 1 cm^2^ [157]. Recently, by growing SnO_2_ on the cross-stacked super-aligned CNTs layers (2.5 µm thick), Luo et al. achieved a hybrid electrode that exhibited enhanced charge extraction and lower recombination rate, leading to an efficiency of 10.5% combined with eliminated hysteresis and excellent stability [156]. In addition, the inferior device performance of carbon-based devices could also be ascribed to that the charge transporting layer is developed for metal-based devices and is not suitable for the application in carbon-based devices. Thus, Chu et al. designed P3HT/graphene composite HTL to enhance hole transportation and achieved a PCE of 12.4% for carbon FPSCs [159].

### Metal Foil Substrates

Thicker metal foil (over 100 μm) could directly be employed as both substrate and electrode. Metal foil is a viable substitute to polymer/ITO substrate, offering advantages such as low sheet resistance, good mechanical durability, and low cost by avoiding the use of expensive ITO electrode. Besides, due to the inherently high heat resistance of metal foil substrate, the high-temperature annealing process of inorganic charge transporting layer can be carried out directly on the substrate. Moreover, the metallic materials have excellent blocking properties due to the easily formed dense metal oxide film, and some charge transport materials like TiO_x_, CuI, which derive from metal foil electrodes could be grown in situ [59, 78, 98, 121]. However, the FPSCs based on metal foil electrodes generally require illumination through the transparent top electrode due to the opaque nature of thick metal foil.

Titanium (Ti) foil, which has been adopted as the electrode in dye-sensitized solar cells earlier [160], is a promising metal electrode in the application of low-cost and large-area FPSCs. There are various methods to prepare the charge transport layer directly on the Ti foil since its oxide form TiO_2_ or TiO_x_ can act as an effective ETL, such as electrochemical anodization and hydrothermal method, besides the traditional spin coating and spray pyrolysis [59, 78, 98]. In 2015, Lee et al. employed a 127 μm thick Ti foil as the substrate and bottom electrode, while an ultrathin Ag film is the transparent top electrode in ITO-free FPSCs. With the CH_3_NH_3_PbI_3_ layer sandwiched between the high-temperature annealed TiO_2_ layer and Spiro-OMeTAD layer, the Ti-based FPSCs exhibited a PCE of 6.15% and maintained 98.5% of initial values after 100 bending cycles at a bending radius of 6 mm [141]. Troughton et al. reported an ITO-free FPSC with an efficiency of 10.3% by using a 150 μm thick Ti foil as the bottom electrode and supporting substrate with nickel mesh laminated above as the transparent top electrode. They indicate that the secondary compact TiO_2_ layer formed during the heat treatment can act as an effective ETL and slow the recombination rate than their glass counterparts [135]. Polished metal substrates with high surface smoothness are favorable for high-quality function layers. Lee et al. demonstrated electropolished Ti foils as the substrate and spray-coated AgNWs as the transparent top electrode in fabricating indium-free FPSCs [105].

Furthermore, there are researches that utilize in-situ growth methods to synthesize TiO_2_ ETLs on Ti foils, which are facile and scalable. Wang et al. demonstrated a PCE of 8.31% for Ti-based FPSCs by adopting electrochemical anodized Ti nanotube arrays as both ETL and perovskite deposition scaffolds. Simultaneously, the CNTs films which are synthesized by floating catalyst chemical vapor deposition play the role of transparent hole-collecting electrode [78]. Recently, Heo et al. prepared a dense TiO_2_ layer on the polished Ti foils by electrochemical anodization and realized a high PCE of 15%, and over 70% of PCE was retained after 1000 bending cycles at a radius of 4 mm [98]. Besides, Xiao et al. employed TiO_2_ nanowires on the Ti foil by a facile hydrothermal method, which offered excellent electron transportability for the straight path to rapidly collect electrons in FPSCs [59].

Except for Ti foils, there are other metal substrates applied like copper foils and stainless foils. However, they all turned out to be less effective. Flexible Cu foils provide advantages such as cost-effectiveness, lightweight and the approaching work function with ITO when being applied as the supporting substrate and electrode in FPSCs. More importantly, Cu-based materials such as CuI, Cu_2_O and CuSCN that possess high hole mobility and low fabrication cost, are potential candidates to replace expensive organic hole transport materials. Nejand et al. introduced Cu as conductive flexible substrates and CuI HTL via iodination, resulting in an efficiency near 13% for long-term durable FPSCs combined with AgNWs as the transparent top electrode [121].

Section 3 has reviewed flexible opaque electrodes used in FPSCs including metal films, opaque carbon materials and metal foils. Their electrical properties and device performance are reviewed in this section. Moreover, we discussed the critical issues of their application in FPSCs and reviewed the corresponding solutions. Metal film electrode and opaque carbon electrode both have their advantages and disadvantages, and it is hard to tell the winner. Metal film electrodes are promising in high-efficiency FPSCs due to their high electrical conductivity and favorable energy alignment. But metal films suffer from long-term stability issues. The diffusion of halide ions and metal atoms, especially under thermal stress or sun illumination, could seriously damage device efficiency. In comparison, opaque carbon electrodes are favorable to achieving highly stable FPSCs, owing to the superior chemical and moisture stability. However, the severe energy loss caused by high sheet resistance and poor interfacial contact with adjacent functional layer, results in inferior efficiency for carbon-based devices than metal electrodes. In addition, the solution-processable carbon paste electrodes also demonstrate the advantage of low fabrication cost, while metal films are expensive since the preparation process is usually under vacuum conditions.

## Summary and Future Perspectives

For the successful realization of durable and efficient FPSCs, it is essential to develop flexible electrodes with reliable mechanical robustness and high electrical conductivity. In this review, convincing candidates of flexible electrodes in FPSCs are assessed from the views of fabrication techniques and physical properties including optoelectrical performance, mechanical flexibility, chemical stability, surface smoothness, and so on. The advantages and disadvantages of various flexible transparent electrodes are listed in Table 2. The future direction and difficulties of flexible electrodes are summarized below:

**Table 2:** Comparision of different flexible transparent electrodes

Flexible transparent electrodes	Advantages	Disadvantages
ITO	Broad optical clarity; high conductivity	Fragile; high fabrication cost; scarce indium supply
PEDOT:PSS	Solution processability high flexibility	Low conductivity; low chemical stability
CNT	Abundant carbon resources; excellent flexibility, transparency; high chemical stability; good moisture resistance	Low conductivity; poor adhesion
Graphene	Abundant carbon resources; excellent flexibility, transparency; high chemical stability; good moisture resistance; low surface roughness	Low conductivity; low doping stability; poor adhesion
AgNWs	Solution processability; excellent conductivity; high transparency	High surface roughness; low chemical stability; poor adhesion
Metal meshes	Excellent conductivity; high transparency	High surface roughness; low chemical stability; complex preparation process

- **(1) Optoelectrical properties** To realize high-efficiency flexible devices, high electrical conductivity and good charge transfer at interface are of great importance for charge transfer and collection. Generally, metallic electrodes are more advantageous due to their low sheet resistance and favorable energy level alignment, while PEDOT:PSS and carbon electrodes demonstrate moderate electrical conductivity. Further doping and post-treatment are required to improve their electrical performance. For window electrodes which need to introduce light into the device, high optical transparency is critical to ensuring light utilization. Among various transparent electrodes, CNTs and graphene electrodes exhibit excellent optical clarity, while metal nanowires and metal meshes demonstrate inferior transparency due to the reflection of incident light. In addition, the light scattering of AgNWs film is beneficial for light harvesting in solar cells. More efforts are needed to fabricate flexible electrodes with both high electrical conductivity and light transmittance to achieve efficient FPSCs and bridge the gap between flexible and rigid devices.
- **(2) Mechanical robustness** The previous reports have concluded that electrodes, especially ITO electrodes, play a significant role in realizing highly robust FPSCs. Therefore, flexible electrodes are required to be flexible and resilient against bending and deforming. The brittle ITO electrode in FPSCs would generate cracks during repeated bending process, which is damaging to device performance. Reducing the thickness of ITO electrode is beneficial for obtaining higher mechanical flexibility. Moreover, TCO-free FPSCs by replacing ITO electrode with other flexible materials are feasible to sustain mechanical stress during device deformation. PEDOT:PSS, carbon electrodes and metallic electrodes with excellent mechanical flexibility are appealing in realizing robust FPSCs. However, AgNWs, metal meshes, CNTs, and graphene electrodes suffer from peeling problems under small radius bending due to the weak adhesion force between electrode and adjacent layers. Coating a capping layer or inserting a film that can form chemical bonding is effective to reinforce the adhesion of electrodes. Besides, structure optimization by reducing the thickness of electrodes or substrates is also beneficial for realizing highly robust FPSCs.
- **(3) Stability** In addition to improving the efficiency and flexibility of FPSCs, the stability of flexible electrodes should be evaluated from the commercialization perspective. Encapsulation is efficient to improve device stability by isolating oxygen and moisture invasion, but it cannot avoid the decomposition within the device. The chemical reaction and ion diffusion between metal electrode and perovskite would cause severe device deterioration as discussed in Sects. 2.4 and 3.1. Various protective layers such as TCO, conducting polymer and carbon materials are introduced as protective layers to improve their stability by isolating perovskite with unstable electrodes. In addition, ITO and PEDOT:PSS electrodes also suffer from chemical and environmental stability problems, and further studies are needed to avoid such adverse effects. In conclusion, the most reliable strategy to solve the stability problem is to replace the corrosive electrode with a carbon electrode, which is chemically inert and water-resistant.
- **(4) Cost** Due to the potential for customized and integrated devices, FPSCs are promising in versatile applications. Cost is taken as the first priority for future commercialization. Thus, the cost of flexible electrodes, which mainly involves raw materials cost and manufacture cost, should be assessed. The most popularly used bottom and top electrodes in FPSCs are ITO and metal films, respectively. Insufficient indium supply and expensive Au, Ag materials raise the material cost of flexible electrodes. In comparison, the abundant carbon resource makes carbon materials (transparent CNTs and graphene, as well as opaque carbon materials) attractive in realizing low-cost flexible devices. Besides, replacing Au and Ag films with cheap Cu film can effectively lower manufacturing cost. In addition to materials cost, high vacuum conditions and high-temperature annealing processes during the fabrication of ITO film and metal films will further increase the manufacturing cost. And metal mesh electrodes are usually prepared by costly or time-consuming techniques, such as photolithography and nano-imprinting. Additionally, the complex transfer and lamination procedures to fabricate CNTs and graphene electrodes are incompatible with continuous roll-to-roll manufacturing. Solution-processable electrodes such as PEDOT:PSS, AgNWs, and carbon pastes are promising for low-cost and large-scale manufacturing. Novel fabrication strategies need to be developed to enable scalable fabrication with roll-to-roll process of flexible electrodes for commercialization.
  In conclusion, it is hard to conclude the best performed flexible electrodes in FPSCs due to the varied perovskite composition and device architecture in literature. Among the flexible transparent electrodes, ITO film is still the most abundantly used electrode in the advanced FPSCs with the highest efficiency. However, the high cost and the brittle nature of ITO electrode could not meet the challenges in broadening the applications of FPSCs. The conductive polymer PEDOT:PSS exhibits outstanding mechanical resilience and good optoelectronic properties, but has concerns in conductivity and chemical stability. CNTs and graphene film are competitive counterparts to ITO electrode and exhibit excellent optical transparency, chemical inertness and mechanical flexibility while inferior electrical conductivity. On the contrary, metal-based flexible electrodes show excellent electrical performance. But the inferior surface morphology and poor chemical stability of metal electrodes are detrimental to the growth of high-quality perovskite film and the long-term stability of device.
  Thus, we believe composite electrodes will be the best solution for flexible transparent electrode. By combining different substitutions, the limitations for single material could be overcome and superior performance in terms of optoelectrical properties, mechanical flexibility and chemical stability can be realized in flexible electrode. For example, metal nanowires or metal meshes combined with a protective layer such as PEDOT:PSS, CNTs or graphene film, cannot only provide better electrical performance, but also act as a passivation and planarization layer to enhance device stability and improve the quality of the functional layer. For flexible opaque electrodes, metal films are effective in realizing high-efficiency FPSCs but have problems in long-term stability and low-cost fabrication. Considering the requirements in developing cost-effective and solution-processable flexible electrodes, carbon-paste electrode is appealing as flexible opaque electrode, but more efforts need to be devoted to solving the interfacial contact problem.
  Except for the above challenges, FPSCs also suffer from large-area fabrication and toxicity issues. With the effective area getting larger, the increase of film defects and series resistance of functional layer will inevitably lead to efficiency loss for FPSCs. Therefore, further research on new compatible methods to reduce the energy loss of large-area FPSC is needed. High-performance FPSCs are based on lead-containing perovskites, and lead leakage from damaged devices can contaminate the environment. In addition to pursuing lead-free devices, proper encapsulation is more feasible in FPSCs and can provide high efficiency while preventing lead leakage. Further improvement and encouraging research on flexible electrodes and FPSCs are underway, and we believe that highly performed flexible electrodes will facilitate the commercialization of FPSCs.
